# Influence of Drying Method on NMR-Based Metabolic Profiling of Human Cell Lines

**DOI:** 10.3390/metabo9110256

**Published:** 2019-10-31

**Authors:** Irina Petrova, Shenyuan Xu, William C. Joesten, Shuisong Ni, Michael A. Kennedy

**Affiliations:** Department of Chemistry and Biochemistry, Miami University, Oxford, OH 45056, USA; petrovi@miamioh.edu (I.P.); xus2@miamioh.edu (S.X.); joestewc@miamioh.edu (W.C.J.); nis@miamioh.edu (S.N.)

**Keywords:** metabonomics, metabolomics, metabolic profiling, NMR, nuclear magnetic resonance spectroscopy, cell line, human cell line, MiaPaCa-2, Panc-1, AsPC-1

## Abstract

Metabolic profiling of cell line and tissue extracts involves sample processing that includes a drying step prior to re-dissolving the cell or tissue extracts in a buffer for analysis by GC/LC-MS or NMR. Two of the most commonly used drying techniques are centrifugal evaporation under vacuum (SpeedVac) and lyophilization. Here, NMR spectroscopy was used to determine how the metabolic profiles of hydrophilic extracts of three human pancreatic cancer cell lines, MiaPaCa-2, Panc-1 and AsPC-1, were influenced by the choice of drying technique. In each of the three cell lines, 40–50 metabolites were identified as having statistically significant differences in abundance in redissolved extract samples depending on the drying technique used during sample preparation. In addition to these differences, some metabolites were only present in the lyophilized samples, for example, n-methyl-α-aminoisobutyric acid, n-methylnicotimamide, sarcosine and 3-hydroxyisovaleric acid, whereas some metabolites were only present in SpeedVac dried samples, for example, trimethylamine. This research demonstrates that the choice of drying technique used during the preparation of samples of human cell lines or tissue extracts can significantly influence the observed metabolome, making it important to carefully consider the selection of a drying method prior to preparation of such samples for metabolic profiling.

## 1. Introduction

Metabonomics is a metabolic profiling technique first described in 1999 [1]. Metabonomics studies, which can be used for biomarker identification for early disease detection and drug development, are accomplished by examining the differences in metabolic profiles of biological fluids between control samples and samples obtained under some form of transformative change or intrinsic difference such as the presence, progression or treatment of disease [2,3,4,5]. Metabonomics has been employed in food science to assess food quality and in nutrition studies to identify diet–health relationships by examination of dietary biomarkers [6,7,8,9,10], in fields such as toxicology and immunology to understand the mechanisms of chemical toxicity and viral infections [11,12], to determine how the use of oral antibiotics alters the gut microbiome based on analysis of fecal extracts [13,14], and to assess the consequences of different forms of acute kidney injury [15,16,17], among many other applications. A recent overview of applications of metabonomic studies was published by Liu and Locasale in 2017 [18]. Metabonomics research has grown exponentially since its inception in 1999, as can be appreciated by considering the number of publications per year determined from a recent literature search of the PubMed citation database (www.ncbi.nlm.nih.gov/pubmed) with the search terms “metabonomics OR metabolomics” specified in the “Title/Abstract” field, which returned over 44,000 publications overall and more than 3000 publications in 2018 alone. Biological fluids, cell line cultures and tissue extracts are common samples used in metabonomics analyses [3,14,16,19,20,21]. Metabolic profiling studies of human cancer cell lines in particular have played a major role in drug discovery as they allow for comparatively inexpensive high-throughput screening to reveal potential drug targets and to study metabolic responses to various stimuli [21,22,23,24]. Metabonomics studies in general can either be targeted, with a focus on specific metabolites, or untargeted where an entire metabolome is analyzed in order to identify and quantify changes in all measurable metabolites [25]. Mass spectrometry (MS) coupled to gas chromatography (GC-MS) or liquid chromatography (LC-MS) and nuclear magnetic resonance spectroscopy (NMR) are the most common analytical techniques used for metabolic profiling [26,27].

Cell lines have been used as models for studying human diseases and metabolic pathways since as early as 1956 [28]. As with metabonomics research itself, the number of metabonomic studies of cell line cultures has also experienced significant growth over the last 15 years, reaching over 400 published studies based on a literature search on the PubMed citation database (www.ncbi.nlm.nih.gov/pubmed) with the search terms “metabonomics OR metabolomics” AND “cell line” OR “cell culture” in the “Title/Abstract” field. Major research areas that employ metabonomics studies of human cell cultures include investigations of many human diseases, pharmacology, systems biology and toxicology [29,30,31,32]. Tumor cells have also been valuable for studying metabolic pathways involved in the biology of cancer and modeling of cancerous formations [33,34,35,36]. In the area of metabolic profiling-based cancer research, human cancer cell line cultures have been utilized in studies of breast cancer, bladder cancer, prostate cancer, oral cancer, brain cancer and pancreatic cancer, among others [37,38,39,40,41,42]. Cell cultures are advantageous in that they represent study systems that are capable of self-replication, thereby providing an infinite amount of material for study, and unlike biological samples that come from animal models and human hosts, cell lines can be easily modified, manipulated and studied in a controlled environment, and used in relatively inexpensive high-throughput screening studies compared to animal model studies [22,43]. Because the cells are cultured in isolation from the original tumor environment and pure cell line cultures, there is a lack of communication between tumor cells and other host and tumor stroma components, which allows for simplified characterization of the tumor cells; however, the lack of the presence of a tumor stroma is limiting, since it participates in formation, maintenance of tumors, cancer cell differentiation and cancer development [36]. Consequently, because of the absence of a biologically relevant tumor stroma context, and due to the absence of interaction with an intact immune system, human cancer cells culture studies cannot perfectly replicate cancer cell metabolism expected in intact tumors in vivo [23,36]. 

Metabolic profiling of human cell cultures typically follows a five-step procedure post cell culture, including sample preparation and processing, data collection, data analysis, identification of metabolites, and determination and analysis of metabolic pathways to which identified metabolites belong [43]. It has been previously reported that the choice of technique used for sample extraction or cell lifting can have an impact on the observed metabolome of the cell culture [44,45,46,47,48,49,50,51]. In light of this fact, it has been suggested that the sample preparation protocols and minimum reporting information should be standardized to provide consistent and accurate results in metabolomic studies [29,48,49,50,52,53,54]. One of the steps that has not been given much attention in the literature up to this point is the influence of the method used to dry the extracts prior to preparation of the concentrated cell extract samples for analysis [29,55,56]. The two most widely used methods of drying cell and tissue extracts are speed-vacuum and lyophilization [29,55,56,57]. In speed-vacuum (SpeedVac) drying, the extract is centrifuged and the solvent evaporated under vacuum at (typically unregulated) room temperature. In lyophilization (freeze-drying), the sample is first frozen with liquid nitrogen, followed by sublimation of the solvent, a process in which the solvent evaporates from its frozen solid state without going through a liquid phase. The temperature inside the trap chamber of a lyophilizer is typically –40 °C to –50 °C and the sample remains frozen throughout the drying process, while temperature inside the speed-vacuum chamber ranges anywhere between 25 °C and 35 °C and, as stated above, is generally unregulated. The vacuum levels in a freeze-dryer typically range between 60 mTorr to 300 mTorr, whereas the vacuum level in a typical SpeedVac is around 10 Torr. Although the relative performance of drying techniques in metabonomics studies has been raised before [57], the issue has not been examined considering the preparation of human cell line extracts with all other procedural steps being the same in the comparison of the two drying techniques. 

Here, we investigated how the observed metabolome of three human pancreatic cancer cell lines, MiaPaCa-2, Panc-1 and AsPC-1, was influenced by application of drying either by SpeedVac or lyophilization. Ten replicate extract samples from each cell line were dried using each technique, and the metabolic profiles of the dried sample extracts were measured by NMR spectroscopy. The resulting metabolic profiles were compared to determine the influence of the choice of drying technique on the observed metabolic profiles. 

## 2. Results

### 2.1. Analysis of MiaPaCa-2 Cells

#### 2.1.1. Representative NMR Data

**Figure 1:**
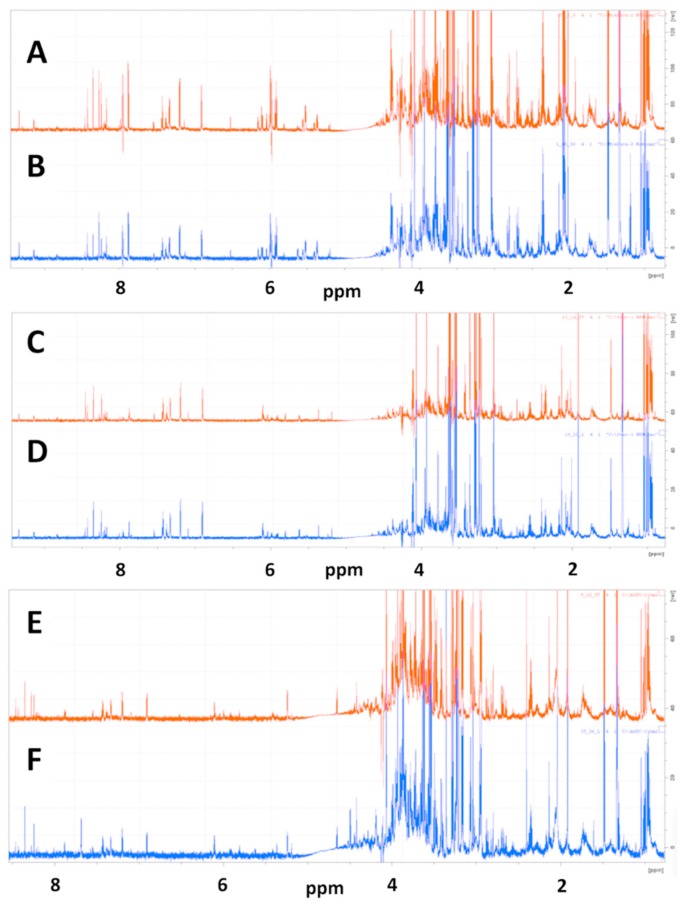
Representative 1D ^1^H NMR spectra of SpeedVac dried and lyophilized MiaPaCa-2, Panc-1 and AsPC-1 cell extracts. 1D CPMG NMR spectra are shown for representative SpeedVac-dried cell extracts for (**A**) MiaPaCa-2, (**C**) Panc-1 and (**E**) AsPC-1 cells and for representative lyophilized cell extracts for (**B**) MiaPaCa-2, (**D**) Panc-1 and (**F**) AsPC-1 cells.

Representative 1D NMR spectra of samples prepared from redissolved speed vacuum dried and lyophilized cell extracts of MiaPaCa-2 cells used for metabolic profiling analysis are shown in Figure 1 with the spectrum of a SpeedVac-dried sample shown in Figure 1A and the spectrum of a lyopilized sample shown in Figure 1B. 

#### 2.1.2. Unsupervised Principal Component Analysis (PCA) and Partial Least Squares-Discriminant Analysis (PLS-DA) of MiaPaCa-2 Cells

**Figure 2:**
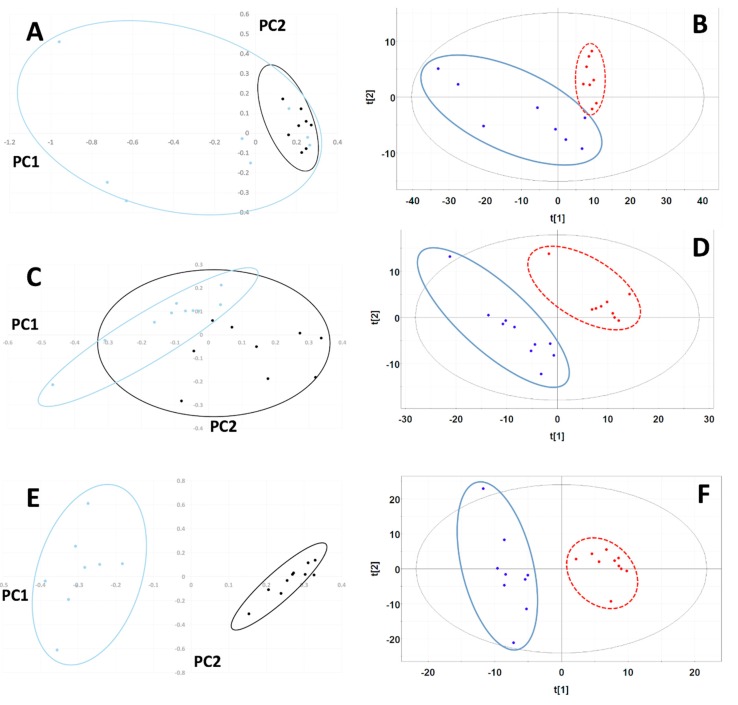
Principal Component Analysis (PCA) and Partial Least Squares-Discriminant Analysis (PLS-DA) scores plots for MiaPaCa-2, Panc-1 and AsPC-1 cell line comparisons. PCA scores plots using the first two principal components for comparisons between lyophilized (blue) and SpeedVac (black) samples from (**A**) MiaPaCa-2, (**C**) Panc-1 and (**E**) AsPC-1 cells. The 95% confidence intervals of the Hotelling’s T^2^ distributions are indicated by the overlaid ovals. PLS-DA scores plots computed using the first two principal components for (**B**) MiaPaCa-2, (**D**) Panc-1 and (**F**) AsPC-1 cell comparisons. Scores indicating lyophilized samples are colored blue and encircled by a solid blue oval line. Scores indicating SpeedVac samples are colored red and encircled by a dashed red line. The 95% confidence intervals of the Hotelling’s T^2^ distributions are indicated by the overlaid black solid oval lines.

A total of 407 manually defined spectral buckets were included in the analysis of MiaPaCa-2 cells. The PCA scores plot for MiaPaCa-2 samples showed significant cluster separation at 95% confidence interval based on an F-test analysis (Mahalanobis distance = 1.35, F-statistic = 3.38, F-critical = 2.10) (Figure 2A) with 87% of the variance accounted for by the first two principal components (PCs) (PC-1 72%, PC-2 15%). Based on a Welch’s t-test, four buckets out of 407 were statistically significant based on the Bonferroni-corrected critical alpha value (0.0001229), and 173 buckets had p-values less than 0.05, which are depicted in the PCA loadings plot in Figure S1A.

The PLS-DA scores plot for MiaPaCa-2 also exhibited statistically significant separation between data set clusters based on the F-test (Mahalanobis distance = 3.75, F-statistic = 26.17, F-critical = 2.10) (Figure 2B). PLS-DA cross-validation revealed an excellent fit with the model (R^2^ = 0.95) and good predictive power (Q^2^ = 0.75) using three PCs. In the PLS-DA, 120 buckets out of 407 had variable importance in projection (VIP) scores greater than 1.0, indicating that these buckets carried the most weight in being responsible for cluster separation.

#### 2.1.3. Prominent Differences in Metabolite Abundances Depending on Drying Method

**Figure 3:**
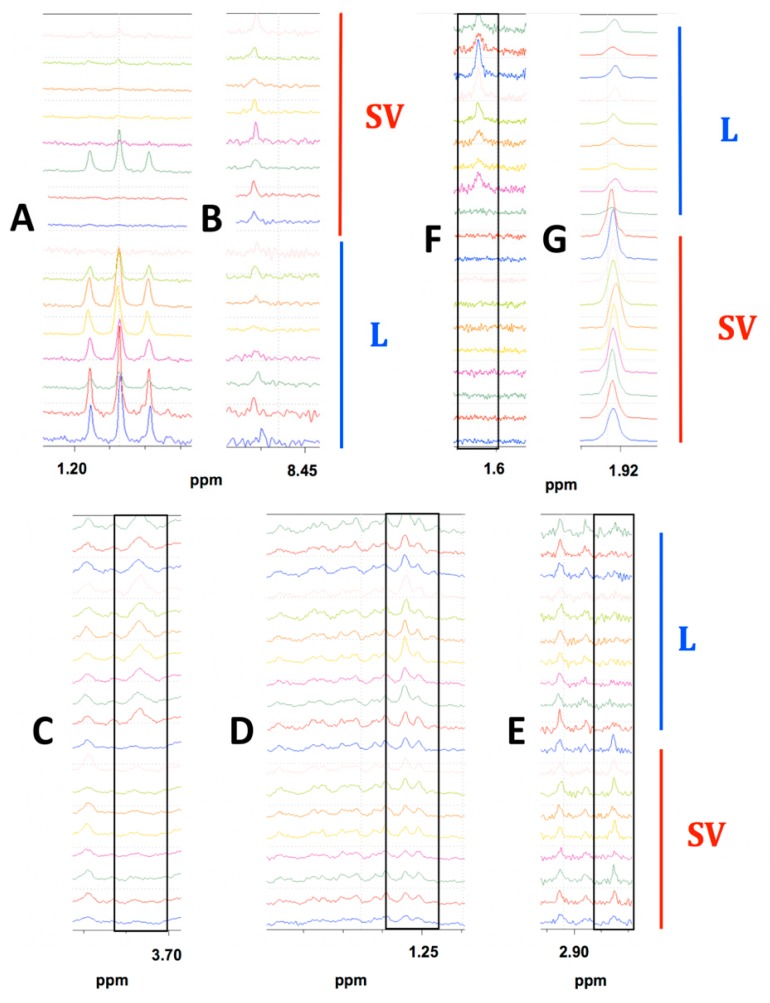
Highlighted spectral differences observed in MiaPaCa-2, Panc-1 and AsPC-1 cell extract comparisons depending on the drying method used for sample preparation. (**A**) Ethanol present in the lyophilized MiaPaCa-2 samples only. (**B**) Formic acid present in both groups of MiaPaCa-2 cell extracts but at higher concentration in SpeedVac samples. The bottom eight spectra were lyophilized samples (L) and the top eight spectra were SpeedVac samples (SV). (**C**) Leucine present only in the lyophilized Panc-1 samples. (**D**) Isoleucine present in both groups of Panc-1 cell extracts but at a higher abundance in the lyophilized samples. (**E**) Trimethylamine present only in SpeedVac dried Panc-1 samples. The bottom nine spectra were SpeedVac samples (SV) and the top 10 spectra were lyophilized samples (L). (**F**) N-methyl-α-aminobutyric acid present only in the lyophilized AsPC-1 samples. (**G**) Acetate was present in both groups but at a higher concentration in SpeedVac samples. The bottom nine spectra were SpeedVac (SV) samples and the top 10 spectra were lyophilized samples (L). Peaks of interest are boxed in each comparison of stacked spectra.

To be considered significant, a bucket had to have either a *p*-value <0.05 or an AUC >0.70 [58]. In MiaPaCa-2 cells, 53 (Table S1) metabolites out of a total of 74 metabolites identified (Table S2) were present at significantly different abundances depending on drying technique. Out of these 53 metabolites, one metabolite was observed only in the lyophilized samples, zero metabolites were only observed in SpeedVac samples, and 52 metabolites were present in both samples, but at different abundances depending on the drying technique used. Figure 3 shows examples where the differences were apparent from visual comparison of the spectra from each MiaPaCa-2 drying group. Ethanol was detected predominantly in the lyophilized samples (Figure 3A) and made a considerable contribution to the PLS-DA cluster separation between the drying techniques as it had the four highest VIP scored buckets. The absence of metabolites such as ethanol in SpeedVac-dried samples was potentially due to more rapid evaporation at room temperature due to higher vapor pressure at this temperature. On the other hand, formic acid was present in both samples but at a higher abundance in those dried by speed-vacuum (Figure 3B). The absence of metabolites such as formic acid in the lyophilized samples may be due to chemical conversion during speed-vacuum drying at a higher temperature. In addition to the different abundances of the identified metabolites in the MiaPaCa-2 extract samples, an additional 54 buckets were identified that had statistically significant differences in intensities between groups based on at least one of our four criteria depending on the drying technique used to prepare the samples, but could not be assigned to a specific metabolite (Table S3) due to limitations of existing databases. An NMR spectrum of the DMEM media was also collected to determine if carry over from the media influenced the profile of metabolites identified from cell extracts (Figure S2A). Of the 19 metabolites identified in the DMEM media (Table S4), 11 were also detected in cell extracts, but eight were not detected in cell extracts, indicating the 11 metabolites detected in the cell extracts originated from the cells and not from carryover from the media since eight metabolites present in the media were absent from the cell extracts.

### 2.2. Analysis of Panc-1 Cells

#### 2.2.1. Representative NMR Data

Representative 1D NMR spectra of samples prepared from redissolved speed vacuum dried and lyophilized Panc-1 cell extracts used for metabolic profiling analysis are shown in Figure 1 with a spectrum of a SpeedVac sample shown in Figure 2C and a spectrum of a lyophilized sample shown in Figure 2D. 

#### 2.2.2. Unsupervised Principal Component Analysis (PCA) and Partial Least Squares-Discriminant Analysis (PLS-DA) of Panc-1 Cells

A total of 359 manually defined spectral buckets were included in the analysis of Panc-1 cells. The PCA scores plot for Panc-1 exhibited significant cluster separation (Mahalanobis distance = 3.11, F-statistic = 21.51, F-critical = 1.95) (Figure 2C) with 74% of the variance accounted for by the first two PCs (PC-1 51%, PC-2 23%). Based on a Welch’s t-test analysis, 18 buckets out of 359 were statistically significant based on the Bonferroni-corrected critical alpha value (0.0001393), which are depicted in the PCA loadings plot in Figure S2B, and 142 buckets had p-values less than 0.05. 

The PLS-DA scores plot for Panc-1 showed statistically significant separation between data sets based on F-test analysis (Mahalanobis distance = 8.47, F-statistic = 159.95, F-critical = 1.95) (Figure 2D). Cross-validation of PLS-DA using three PCs indicated excellent fit of the data to the model (R^2^ = 0.98) and high predictive capability of the model (Q^2^ = 0.90). In the PLS-DA, 160 buckets out of 359 had VIP scores greater than 1.0, indicating that these buckets carried the most weight in being responsible for cluster separation.

#### 2.2.3. Prominent Differences in Metabolite Abundances Depending on Drying Method

In Panc-1 cells, 49 metabolites (Table S5) out of a total of 63 identified (Table S6) were present at significantly different abundances depending on drying technique. Out of these 49 metabolites, two metabolites were observed only in the lyophilized samples, two metabolites were only observed in SpeedVac samples, and 47 metabolites were present in both samples, but at different abundances depending on the drying technique used. Figure 3C–E demonstrates differences in metabolite abundances that are visually apparent between drying groups for Panc-1 sample extracts. Leucine was detected only in the lyophilized samples (Figure 3C) and had considerable contribution towards differences between the drying techniques as it had the highest VIP bucket. Isoleucine was present in both samples but at a higher abundance in those dried by lyophilization (Figure 3D). Trimethylamine was detected only in speed-vacuumed samples (Figure 3E). The absence of metabolites such as trimethylamine in the lyophilized samples was potentially due to chemical degradation. Trimethylamine was most likely converted from glycerophosphocholine which was at a higher abundance in the lyophilized samples. In addition to the different abundances of identified metabolites in Panc-1 extract samples prepared by different drying techniques, there were an additional 35 buckets that had statistically significant differences in intensities, based on at least one of our four criteria, depending on the drying technique used to prepare the samples that could not be assigned to a specific metabolite (Table S7). Based on the analysis of the NMR spectrum of the DMEM media (Figure S2A), of the 19 metabolites identified in the DMEM media (Table S3), 11 were also detected in cell extracts, but eight were not detected in cell extracts, indicating the 11 metabolites detected in the cell extracts originated from the cells and not from carryover from the media since eight metabolites present in the media were absent from the cell extracts. 

### 2.3. Analysis of AsPC-1 Cells

#### 2.3.1. Representative NMR Data

Representative 1D NMR spectra of samples prepared from redissolved speed vacuum dried and lyophilized AsPC-1 cell extracts used for metabolic profiling analysis are shown in Figure 1, with a spectrum of a SpeedVac sample shown in Figure 1D and a spectrum of a lyophilized sample shown in Figure 1E.

#### 2.3.2. Unsupervised Principal Component Analysis (PCA) and Partial Least Squares-Discriminant Analysis (PLS-DA) of AsPC-1 Cells

A total of 307 manually defined spectral buckets were included in the analysis of Panc-1 cells. The PCA scores plot for AsPC-1 also displayed clear cluster separation at 95% confidence interval (Mahalanobis distance = 11.80, F-statistic = 310.40, F-critical = 1.95) (Figure 2E) with 85% of the variance accounted for by the first two PCs (PC-1 51%, PC-2 34%). Based on a Welch’s t-test analysis, 21 buckets out of 307 were statistically significant based on the Bonferroni-corrected critical alpha value (0.0001629), which are depicted in the PCA loadings plot in Figure S1C, and 107 buckets had p-values less than 0.05. 

The PLS-DA scores plot for AsPC-1 cell line samples showed separation of the two groups that was statistically significant based on F-test analysis (Mahalanobis distance = 7.64, F-statistic = 130.05, F-critical = 1.95) (Figure 3A). PLS-DA cross-validation using two PCs (Figure 2F) generated R^2^ = 0.80 indicating excellent data agreement with the model, and Q^2^ = 0.94, indicating great model predictive power. In the PLS-DA, 113 buckets out of 307 had VIP scores greater than 1.0, indicating that these buckets carried the most weight in being responsible for cluster separation.

#### 2.3.3. Prominent Differences in Metabolite Abundances Depending on Drying Method

In AsPC-1 cells, 44 metabolites (Table S8) out of a total of 60 identified (Table S9) were present at significantly different abundances depending on drying technique. Out of these 44 metabolites, four metabolites were observed only in the lyophilized samples, zero metabolites were only observed in SpeedVac samples, and 40 metabolites were present in both samples, but at different abundances depending on the drying technique used. Figure 3F–G displays visual differences between each group by comparing spectra from each AsPC-1 sample. N-methyl-α-aminobutyric acid was detected only in the lyophilized samples (Figure 3F). Acetate was found in both samples but at a higher concentration in those dried by speed-vacuum (Figure 3G). The absence of metabolites such as acetate in the lyophilized samples was likely due to chemical conversion at the relatively high temperature compared to lyophilization during speed-vacuum drying. In addition to the different abundances of identified metabolites in AsPC-1 extract samples prepared by different drying techniques, an additional 60 buckets that had statistically significant differences in intensities based on at least one of our four criteria depending on the drying technique used to prepare the samples could not be assigned to a specific metabolite (Table S10). An NMR spectrum of the RPMI media was also collected (Figure S2B) to determine if carry over from the media influenced the profile of metabolites identified from cell extracts. Of the 22 metabolites identified in the RPMI media (Table S11), nine were also detected in cell extracts, but 13 were not detected in cell extracts, indicating the nine metabolites detected in the cell extracts originated from the cells and not from carryover from the media since 13 metabolites present in the media were absent from the cell extracts. 

## 3. Discussion

The data presented here indicate that the choice of drying technique used to prepare extracts from human cell lines for metabolic profiling has a significant impact on the observed metabolic profiles. 70–75% of all the buckets intensities were significantly different depending on the drying technique in the observed metabolome in each of the three cell lines. In the three human pancreatic cancer cell lines investigated in this study, the number of metabolites whose abundances were significantly different depending on drying technique ranged from 44 in AsPC-1 cell extracts to 53 in MiaPaCa-2 cell extracts. A prominent difference in the metabolic profiles depending on drying technique was that some metabolites were present in samples prepared by one drying technique but completely absent in the other (Table 1). For instance, n-methyl-α-aminoisobutyric acid was present in the lyophilized samples but undetected in speed-vacuum dried samples in cell extracts prepared from all three cell lines. Other metabolites detected in lyophilized cell extracts but undetected in SpeedVac samples included 3-hydroyvaleric acid, N-methyl nicotinamide and sarcosine, which was only detected in AsPC1 cells.

On the other hand, one metabolite was present in speed-vacuumed samples but absent in the lyophilized samples (Table 1), i.e., trimethylamine, which was only present in Panc-1 cell extracts prepared by SpeedVac. The absence of trimethylamine in the lyophilized samples could be due to chemical conversion during speed-vacuum drying that takes place at a higher temperature compared to during lyophilization.

The majority of metabolites that were present at different abundances depending on drying technique were present in extracts prepared by both drying techniques (Table 1). Forty-one metabolites were found to be more concentrated in samples dried by speed-vacuum. Fifteen out of 41 metabolites that were present at higher abundances in SpeedVac samples were found only in AsPC-1. The AsPC-1 cell line also had more metabolites present in greater amounts in samples dried by speed-vacuum compared to the MiaPaCa-2 and Panc-1 cells. This could be due to two factors: the differences in cellular composition in cancer cell lines [21] and the different optimal growth media, i.e., RPMI, used to culture the AsPC-1 cells [51]. Sixty-one metabolites were present at higher concentrations in the lyophilized samples. The lower abundances of these metabolites in speed-vacuumed samples was likely caused by more rapid evaporation at room temperature (or higher) during speed vacuum drying compared to freeze-drying, since metabolites generally have higher vapor pressures at higher temperatures, and a higher rate of chemical conversion of heat labile metabolites to different compounds at the higher temperature of the SpeedVac during drying. 

About 10–15% of all significant buckets in each cell line could not be identified from the existing databases (i.e., no matching peaks could be identified in from the existing databases) therefore limiting our ability to draw specific conclusions from this missing fraction of data. While some metabolites were more abundant after SpeedVac drying, lyophilization seems to be the generally superior technique compared to centrifugal evaporation under vacuum as it seemed to preserve the original metabolome of the cell lines to a higher degree based on the general observation of including more metabolites, or the same metabolites at higher concentrations compared to SpeedVac prepared samples.

## 4. Materials and Methods 

### 4.1. Cell Cultures and Preparation of Cell Extracts

MiaPaCa-2, Panc-1, and AsPC-1 were purchased from the American Type Culture Collection (Manassas, VA, USA). MiaPaCa-2 and Panc-1 were grown in high glucose Dulbecco’s Modified Eagle Medium, (DMEM) and AsPC-1 cells were grown in Roswell Park Memorial Institute (RPMI) medium. Both media were supplemented with 10% fetal bovine serum and 1% penicillin–streptomycin. Sixty 100 mm × 15 mm petri dishes per cell line were harvested and extracted as previously described by Watanabe et al. [21]. Briefly, the cells were scraped using chilled phosphate-buffered saline (PBS), washed 3× in cold PBS buffer, and then stored in −80 °C prior to cell extraction. Frozen cells thawed on ice, resuspended in 1.5 mL of ice-cold chloroform-methanol-water (1:1:1) solution and vortexed, after which they were chilled on ice for 15 min. The tubes were then centrifuged for 15 min at 15,000× *g*. The top layer (hydrophilic) and the bottom layer (lipophilic) were separated and transferred to new Eppendorf tubes. Hydrophilic extracts were dried and used for NMR spectroscopic analysis. 

### 4.2. Drying Methods

Thirty samples of each cell line were dried in a speed vacuum centrifugal concentrator (UVS800DA THERMO Savant Sunnyvale, CA). Another thirty samples of each cell line were frozen in liquid nitrogen and dried using a lyophilizer (Labconco 7740020, Kansas City, MO, USA). The drying time used for both SpeedVac and lyophilization were kept constant. MiaPaCa-2 and Panc-1 cells were dried for 21 h, and AsPC-1 for 20 h. For lyophilization, it was ensured that the samples remained frozen throughout the sublimation process and did not thaw into a liquid prior to complete lyophilization. 

### 4.3. Preparation of Samples for NMR Data Collection 

Following resuspension in 150 µL of buffer (150 mM potassium phosphate at pH 7.4, 1 mM trimethylsilyl propionate (TSP), 0.01% sodium azide in 100% deuterium oxide (the buffer was first prepared in 100% H2O, lyophilized, and then reconstituted in 100% D2O)) [21], cell line extracts were combined in the following manner: ten were combined to generate a representative sample for lyophilized samples for 2D NMR experiments needed for confirmation of metabolite assignments, another ten were combined and used as a representative sample for speed vacuumed samples for 2D NMR experiments needed for confirmation of metabolite assignments, and the last forty were used to create ten replicate samples (two samples combined for each NMR sample to increase the extract concentration for NMR analysis) per drying method for 1D NMR experiments. Overall, a total of 60 samples were prepared for 1D NMR spectroscopy analysis. A total of six representative samples were made for analysis using 2D NMR spectroscopy. Samples of both the DMEM and RPMI media were also prepared for NMR analysis as controls.

### 4.4. NMR Data Collection

All NMR experiments were recorded at 850.104 MHz at 298K on a Bruker Avance Spectrometer. 150 microliters of redissolved extracts were transferred to 3 mm NMR tubes for 1D experiments. Two hundred and fifty microliters of redissolved extracts were transferred to 5 mm Shigemi tubes for 2D experiments. One-dimensional ^1^H Carr-Purcell-Meiboom-Gill (CPMG) NMR spectra were collected using a spectral width of 20.0 ppm and 32K points resulting in 1.21 s acquisition time, 512 scans, 4 dummy scans, 1.80 s recycle delay and 1 ms mixing time. Two-dimensional ^1^H-^1^H TOCSY NMR experiments were performed using a spectral width of 10.0 ppm and 2K points, 1.5 s recycle time and 60 ms mixing time [14,17,19,20,21,58,59,60,61]. Two-dimensional ^1^H-^13^C HSQC NMR experiments were performed using non-uniform sampling in the indirect carbon dimension [62,63]. 

### 4.5. Data Analysis

NMR spectra were phase adjusted, baseline corrected, referenced to 0.0 ppm using the internal TSP standard manually using Top-Spin 3.6.1 (Bruker BioSpin, Billerica, MA, USA) and normalized to the total intensity prior to statistical analysis. Manual peak bucketing and principal components analyses (PCA) were performed using AMIX (Bruker BioSpin, Billerica, MA, USA) as we have described previously [21,58,60,61]. PCA scores plot cluster analyses using the Mahalanobis distance and F-test calculations were performed as described previously [61]. Statistical significance analysis of bucket intensity differences were carried out as previously described [60] using a Bonferroni-corrected critical alpha values for p-values calculated using a Welch’s t-test and fold change (FC) were calculated using Excel. Partial least squared-discriminant analysis (PLS-DA), and variable importance in projection (VIP) scores were obtained using the SIMCA-P software (Umetrics, Santorious Stedim, Umeå, Sweden). Area under the curve (AUC) of the receiver operating characteristic (AUROC) was conducted using the MetaboAnalyst (www.metaboanalyst.ca) [17,58].

### 4.6. Metabolite Identification

One-dimensional NMR peaks that were statistically different between the groups were initially and putatively identified using ChenomX Profiler (https://www.chenomx.com/), the Biological Resonance Data Bank (BMRB) [64,65] and the Human Metabolome Database (HMDB) [66,67,68,69]. Two-dimensional NMR data were analyzed using the COLMAR software [70,71] (https://ccic.ohio-state.edu/) to confirm metabolite assignments. The confidence in metabolite assignments was ranked using the RANCM scheme developed in our laboratory [72]. 

## 5. Conclusions

Sample preparation of cell line cultures for metabolomic analyses generally requires a drying step. In the case of metabolic profiling of human cell line cultures, a chloroform/methanol extraction procedure is normally used to isolate the hydrophobic and hydrophilic fractions that must be dried prior to reconstitution in a buffer appropriate for instrumental analysis. Considering absence of standardized procedures for sample preparation for metabolic profiling studies, we examined whether the choice of drying method had any influence on the observed metabolome of hydrophilic extracts measured by NMR of three different human pancreatic cell line cultures. We determined that the metabolic profiles of each cell line differed significantly depending on the drying technique used during sample preparation. Certain metabolites were only found in samples dried by a specific drying technique, suggesting either that some metabolites evaporated during drying by one method and not the other, or that some metabolites went through chemical transformation in one of the sample preparation methods compared to the other. Metabolites undetected in speed-vacuum-dried samples may have been lost due to more rapid evaporation over time due to their having higher vapor pressure near room temperatures compared to at the relatively low temperature of the sample maintained during lyophilization. Overall, the lyophilized samples contained more metabolites at higher concentrations in two out of the three cell lines tested, i.e., MiaPaCa-2 and Panc-1, appearing to make it the technique of choice for the sample drying step during sample preparation. However, the two cell lines that had more metabolites more abundant in the lyophilized samples, MiaPaCa-2 and Panc-1, were grown on the same DMEM media whereas the one cell line that had more metabolites abundant in the SpeedVac samples were cultured using a different media, i.e., RPMI. Therefore, future experiments should examine whether the choice of media used to culture human cell lines for metabolic profiling can influence the efficiency of metabolite recovery using different drying methods and potentially explain the difference in the abundances of metabolites following one drying method over the other. Another aspect of the research that warrants further investigation is simple replication of the reported experiments. For example, in the case of the MiaPaCa-2 samples, the PCA indicates that the lyophilized samples were widely scattered in the PCA eigenspace of the scores plot in comparison to the SpeedVac samples. In contrast, in the AsPC-1 cells, the lyophilized and SpeedVac samples were tightly clustered and well separated in the PCA scores plot. These disparate observations indicated that the experiments should be repeated to determine if these observations are reproducible. Future work could also include working with the database developers to generate more complete databases since approximately half of the observed NMR resonances could not be assigned to a metabolite. Finally, it would be beneficial to repeat the experiment using an MS-based analytical technique for data collection to achieve more comprehensive metabolic profiles. In conclusion, this research demonstrates that the drying technique used in the preparation of human cell culture samples for metabolic profiling can have a significant effect on the observed metabolomes and the selection of the drying technique used during sample preparation should be given careful consideration when designing the sample preparation strategy.

## Figures and Tables

**Table 1:** Summary of metabolite differences in resuspended MiaPaCa-2, Panc-1 and AsPC-1 cell extract samples depending on the drying method used for sample preparation.

Only in Lyophilized	More Abundant in Lyophilized Cell Extracts	More Abundant in Speed-Vacuum Dried Extracts
3-Hydroxyisovaleric acid ^3^	1,3,7-trimethyluric acid1^3^	Acetate ^2,3^
N-methyl-α-aminoisobutyric acid ^1,2,3^	Creatinine1^3^	1,9-Dimethyluric acid ^1,3^
N-Methylnicotinamide ^3^	Methanol1^3^	Cytidine Triphosphate ^3^
Sarcosine ^3^	3-Cresotinic acid ^3^	Glucose-6-phosphate ^3^
Leucine ^2^	Threonine ^3^	Glucose ^3^
Ethanol ^1^	1,3-Dihydroxyacetone ^3^	1-methyladenosine ^1,3^
**Only in Speed-Vacuum**	l-Aspartic acid ^1,3^	Carnosine ^3^
Trimethylamine ^2^	2-phosphoglycerate ^3^	l-Threonine ^3^
	l-Methionine ^1,3^	Tyramine ^3^
	Creatine ^2,3^	Mannose-6-phosphate ^3^
	Glycerophosphocholine ^1,3^	Uracil ^3^
	Butanone ^3^	D-Alanine1^3^
	Methylcysteine ^3^	Glycine ^3^
	Leucine ^1,3^	l-Lactic acid ^3^
	Capric acid ^3^	l-Valine ^3^
	Galactaric acid ^3^	Isoleucine ^3^
	Phosphocreatine ^3^	l-Phenylalanine ^3^
	Pyruvate ^3^	Glycerate ^3^
	Uridine diphosphategalactose ^1,2^	N-Carbamoyl-β-alanine ^3^
	Glucaric acid ^1^	Glycerol ^3^
	Threonic acid ^1^	Trimethylamine ^1^
	Uracil ^1^	Fumaric acid ^1^
	Acetylglycine ^1^	Formic acid ^1,2^
	l-Phenylalanine ^1,2^	1-Butanol ^1^
	creatine phosphate ^1,2^	2-hydroxybutyrate ^1^
	2,3-Diphosphoglyceric acid ^1^	Taurine ^1^
	l-Tyrosine ^1,2^	1-Methyluric acid ^1^
	l-Isoleucine ^1,2^	NAD+^1^
	Uridine ^1^	l-Malic acid ^1^
	Inosine ^1,2^	Methanol ^2^
	S-Adenosylhomocysteine ^1^	l-Aspartic acid ^2^
	1-Butanol ^2^	Uracil ^2^
	S-Adenosylhomocysteine ^1,2^	Acetylcholine ^2^
	Histidine ^2^	l-Methionine ^2^
	Glutathione ^1,2^	Glycerophosphocholine ^2^
	Methionine sulfoxide ^1^	Phosphoenolpyruvic acid ^2^
	Phosphorylcholine ^1^	1-methylguanosine ^2^
	Cellobiose ^1^	3-Methyladipic acid ^2^
	3-Hydroxyisovaleric acid ^1^	Oxypurinol ^2^
	l-Valine ^1,2^	Adenine ^2^
	Pyroglutamate ^1^	Guanosine ^2^
	Acetylphosphate ^1^	
	Glucose ^1,2^	
	Glucose-6-phosphate ^1^	
	Myoinositol ^1,2^	
	1-Methylguanine ^1^	
	1,1-Dimethylbiguanide ^1^	
	Pyruvatoxime ^1,2^	
	l-lactic acid ^1,2^	
	3,7-Dimethyluric acid ^2^	
	Succinic acid ^2^	
	Adenosine phosphosulfate ^2^	
	Mannose 6-phosphate ^2^	
	3-Phosphoglyceric acid ^2^	
	NAD+^2^	
	N-acetylneuraminic acid ^2^	
	Lysine ^2^	
	5-aminolevulinate ^2^	
	Trimethylamine N-oxide ^2^	
	Taurine ^2^	

^1^ MiPaCa-2 cell extracts, ^2^ Panc-1 cell extracts, ^3^ AsPC-1 cell extracts.

## Supplementary Materials

The following are available online at https://www.mdpi.com/2218-1989/9/11/256/s1, Figure S1. PCA Loadings plots for each cell line comparison; Figure S2. 850 MHz ^1^H NMR spectrum of DMEM and RPMI media; Table S1. Identified metabolites whose abundance were significantly different depending on drying technique in the MiaPaCa-2 cell line; Table S2. Complete list of identified metabolites in the MiaPaCa-2 cell extracts; Table S3. Full list of unidentified buckets for MiaPaCa-2 that were determined to be significant; Table S4. List of metabolites identified in the NMR spectrum of DMEM media; Table S5. Identified metabolites whose abundance were significantly different depending on drying technique in the Panc-1 cell line; Table S6. Complete list of identified metabolites in the Panc-1 cell extracts; Table S7. Full list of unidentified buckets for Panc-1 that were determined to be significant; Table S8. Identified metabolites that were found significant for AsPC-1 cell line. Only first significant buckets for each metabolite are shown; Table S9. Complete list of identified metabolites in the AsPC-1 cell extracts; Table S10. Full list of unidentified buckets for AsPC-1 that were determined to be significant; Table S11. List of metabolites identified in the NMR spectrum of RPMI media; Raw NMR data has been archived in the public figshare data repository at https://figshare.com/projects/Influence_of_Drying_Method_on_NMR-Based_Metabolic_Profiling_of_Human_Cell_Lines/68144.
