# Supplementary material for: Influence of Drying Method on NMR-Based Metabolic Profiling of Human Cell Lines
Source: Metabolites. 2019 Oct 31;9(11):256. doi: 10.3390/metabo9110256 (PMC6918379; doi:10.3390/metabo9110256)
Supplement: Supplementary file 1 [file metabolites-09-00256-s001.pdf]

supplementary material for

Influence of Drying Method on NMR-Based Metabolic Profiling of Human Cell Lines

Irina Petrova [1], William C. Joesten [1], Shuisong Ni[1], and Michael A. Kennedy [1,*]

Contents

Table S1. Identified metabolites whose abundance were significantly different depending on drying technique in the MiaPaCa-2 cell line.

Table S2. Full list of unidentified buckets for MiaPaCa-2 that were determined to be significant.

Table S3. Identified metabolites whose abundance were significantly different depending on drying technique in the Panc-1 cell line.

Table S4. Full list of unidentified buckets for Panc-1 that were determined to be significant.

Table S5. Identified metabolites that were found significant for AsPC-1 cell line. Only first significant buckets for each metabolite are shown.

Table S6. Full list of unidentified buckets for AsPC-1 that were determined to be significant.

Table S1. Identified metabolites whose abundance were significantly different depending on drying technique in the MiaPaCa-2 cell line.

ppm	Metabolite Identification	p-value	VIP	Fold Change (Error)	AUC	Rank
1.187	Ethanol	3.29E-03	1.97	-3.63 (2.13)	0.89	1
3.654	Ethanol	8.85E-05	1.92	-2.67 (0.95)	0.98	1
1.179	Ethanol	2.31E-03	1.85	-3.39 (1.97)	0.95	1
1.196	Ethanol	3.21E-03	1.84	-2.94 (1.7)	0.95	1
3.663	Ethanol*	1.41E-04	1.67	-2.06 (0.78)	0.97	1
3.645	Ethanol*	3.07E-03	1.20	-1.36 (0.34)	0.88	1
4.032	Xylulose	2.38E-04	1.75	-1.51 (0.36)	0.97	2
3.663	Xylulose*	1.41E-04	1.67	-2.06 (0.78)	0.97	2
4.038	Xylulose	2.74E-04	1.62	-1.42 (0.36)	0.95	2
4.050	Xylulose*	1.60E-03	1.13	-1.71 (1.24)	0.98	2
3.571	Xylulose	1.03E-02	1.12	-1.41 (0.52)	0.88	2
3.694	Xylulose*	5.66E-03	0.96	-1.63 (1.92)	0.94	2
3.674	Xylulose	8.81E-03	0.92	-1.85 (3.27)	1.00	2
3.680	Xylulose	1.36E-02	0.89	-1.5 (3.35)	0.89	2
3.687	Xylulose*	1.43E-02	0.88	-1.53 (2.2)	0.89	2
4.387	Xylulose*	2.86E-02	0.85	-1.34 (0.94)	0.83	2
4.382	Xylulose*	4.10E-02	0.84	-1.35 (0.99)	0.78	2
4.376	Xylulose*	5.91E-02	0.77	-1.6 (3.11)	0.86	2
3.392	D-Pantethine	3.58E-04	1.68	-2.52 (1.53)	0.97	2
4.003	D-Pantethine*	9.55E-03	0.91	-1.49 (1.72)	0.91	2
3.667	Gluconic acid	1.67E-04	1.45	-2.2 (1.04)	1.00	2
4.139	Gluconic acid*	1.47E-02	0.87	-1.43 (0.78)	0.88	2
3.787	Gluconic acid	3.62E-02	0.79	-1.21 (0.45)	0.81	2
4.104	Lactic acid	3.78E-02	1.45	-1.4 (0.45)	0.83	3
2.002	N-Acetyl-L-Alanine	2.64E-02	1.42	1.22 (0.38)	0.80	2
8.352	Adenosine*	7.18E-01	1.39	-1.05 (0.82)	0.52	2
4.427	Adenosine	1.71E-02	1.02	-1.21 (0.4)	0.86	2
8.276	Adenosine	6.66E-01	1.00	1.07 (0.49)	0.52	2
4.422	Adenosine	4.52E-02	0.99	-1.24 (0.74)	0.81	2
4.451	Adenosine	6.29E-02	0.92	-1.29 (1.02)	0.80	2
4.295	Adenosine*	1.58E-02	0.87	-1.45 (1.16)	0.88	2
3.921	Adenosine*	2.22E-02	0.87	-1.45 (1.92)	0.84	2
3.856	Adenosine*	2.15E-02	0.85	-1.33 (1.14)	0.81	2
4.439	Adenosine	6.49E-02	0.82	-1.42 (2.39)	0.72	2

7.542	Uracil	1.75E-03	1.37	-1.65 (0.39)	0.91	2
3.446	Pantothenic acid	7.12E-03	1.30	1.45 (0.59)	0.84	2
2.410	Pantothenic acid	3.62E-02	0.89	-1.18 (0.34)	0.80	2
3.645	Glycerophosphocholine*	3.07E-03	1.20	-1.36 (0.34)	0.88	2
3.953	Glycerophosphocholine*	1.97E-01	1.00	-1.08 (0.24)	0.66	2
3.659	Glycerophosphocholine	4.38E-03	1.00	-1.68 (1.01)	0.94	2
3.957	Glycerophosphocholine*	5.37E-03	0.98	-1.38 (1.01)	0.94	2
3.694	Glycerophosphocholine*	5.66E-03	0.96	-1.63 (1.92)	0.94	2
3.963	Glycerophosphocholine	5.55E-03	0.96	-1.54 (2.5)	0.94	2
3.949	Glycerophoshocholine*	8.36E-03	0.92	-1.52 (2.33)	0.91	2
3.907	Glycerophosphocholine*	1.49E-02	0.90	-1.46 (1.74)	0.88	2
3.687	Glycerophosphocholine*	1.43E-02	0.88	-1.53 (2.2)	0.89	2
3.921	Glycerophosphocholine*	2.22E-02	0.87	-1.45 (1.92)	0.84	2
3.234	Glycerophosphocholine*	2.42E-02	0.86	-1.7 (2.33)	0.91	2
5.529	UDP-N-Acetylglucosamine	2.93E-02	1.16	-1.34 (0.31)	0.80	2
3.989	UDP-N-Acetylglucosamine*	3.02E-03	1.03	-1.49 (1.16)	0.98	2
4.254	UDP-N-Acetylglucosamine*	1.31E-02	1.03	-1.35 (0.5)	0.88	2
5.992	UDP-N-Acetylglucosamine	5.78E-03	0.97	-1.55 (0.82)	0.91	2
3.827	UDP-N-Acetylglucosamine	8.42E-03	0.94	-1.43 (0.89)	0.89	2
3.890	UDP-N-Acetylglucosamine	1.07E-02	0.92	-1.61 (1.83)	0.91	2
4.003	UDP-N-Acetylglucosamine*	9.55E-03	0.91	-1.49 (1.72)	0.91	2
4.366	UDP-N-Acetylglucosamine*	1.00E-02	0.91	-1.38 (0.87)	0.88	2
4.241	UDP-N-Acetylglucosamine*	1.06E-02	0.90	-1.43 (1.46)	0.88	2
3.821	UDP-N-Acetylglucosamine	1.07E-02	0.90	-1.49 (1.99)	0.88	2
5.520	UDP-N-Acetylglucosamine	1.57E-02	0.88	-1.47 (0.58)	0.86	2
2.089	UDP-N-Acetylglucosamine	1.47E-02	0.88	-1.56 (1.61)	0.89	2
5.986	UDP-N-Acetylglucosamine	1.34E-02	0.87	-1.67 (1.11)	0.91	2
5.967	UDP-N-Acetylglucosamine	1.83E-02	0.87	-1.73 (0.85)	0.86	2
4.295	UDP-N-Acetylglucosamine*	1.58E-02	0.87	-1.45 (1.16)	0.88	2
3.921	UDP-N-Acetylglucosamine*	2.22E-02	0.87	-1.45 (1.92)	0.84	2
3.876	UDP-N-Acetylglucosamine	2.36E-02	0.87	-1.45 (1.93)	0.88	2
5.524	UDP-N-Acetylglucosamine	2.46E-02	0.86	-1.46 (0.7)	0.83	2
4.229	UDP-N-Acetylglucosamine*	2.31E-02	0.85	-1.37 (1.47)	0.88	2
4.387	UDP-N-Acetylglucosamine*	2.86E-02	0.85	-1.34 (0.94)	0.83	2
3.856	UDP-N-Acetylglycosamine*	2.15E-02	0.85	-1.33 (1.14)	0.81	2
3.802	UDP-N-Acetylglucosamine	2.84E-02	0.84	-1.72 (3.63)	0.88	2
4.360	UDP-N-Acetylglucosamine*	2.21E-02	0.84	-1.4 (1.28)	0.81	2
4.382	UDP-N-Acetylglucosamine*	4.10E-02	0.84	-1.35 (0.99)	0.78	2
3.816	UDP-N-Acetylglucosamine	2.63E-02	0.83	-1.62 (3.8)	0.97	2
4.370	UDP-N-Acetylglucosamine	2.61E-02	0.82	-1.47 (1.6)	0.89	2
4.235	UDP-N-Acetylglucosamine*	2.69E-02	0.81	-1.34 (0.92)	0.83	2

5.515	UDP-N-Acetylglucosamine	2.87E-02	0.79	-1.56 (0.96)	0.84	2
5.980	UDP-N-Acetylglucosamine	4.79E-02	0.78	-1.38 (0.57)	0.75	2
4.376	UDP-N-Acetylglucosamine*	5.91E-02	0.77	-1.6 (3.11)	0.86	2
4.050	Myoinositol*	1.60E-03	1.13	-1.71 (1.24)	0.98	3
3.101	Phenylalanine	3.29E-02	1.12	-1.42 (0.84)	0.80	1
3.094	Phenylalanine	5.34E-03	1.07	-1.75 (0.99)	0.97	1
3.989	Phenylalanine*	3.02E-03	1.03	-1.49 (1.16)	0.98	1
7.441	Phenylalanine	1.51E-02	0.99	-1.64 (1.78)	0.83	1
7.390	Phenylalanine	1.70E-02	0.96	-1.59 (2.7)	0.80	1
3.111	Phenylalanine	1.11E-02	0.94	-1.48 (0.83)	0.89	1
7.382	Phenylalanine	1.58E-02	0.91	-1.65 (3.82)	0.80	1
4.003	Phenylalanine*	9.55E-03	0.91	-1.49 (1.72)	0.91	1
3.118	Phenylalanine	1.67E-02	0.91	-1.35 (0.52)	0.83	1
4.506	NAD	4.86E-03	1.11	-1.46 (0.73)	0.91	2
4.512	NAD	4.49E-02	0.82	-1.32 (0.77)	0.84	2
4.518	NAD	3.86E-02	0.82	-1.41 (1.1)	0.84	2
3.771	Argininosuccinic acid*	2.73E-03	1.09	-1.51 (1.18)	0.98	2
6.522	Argininosuccinic acid	3.36E-03	1.05	3.52 (5.03)	0.88	2
4.254	Argininosuccinic acid*	1.31E-02	1.03	-1.35 (0.5)	0.88	2
1.696	Argininosuccinic acid	4.15E-02	0.87	-1.32 (1.08)	0.83	2
1.704	Argininosuccinic acid	3.87E-02	0.87	-1.36 (1.43)	0.83	2
1.726	Argininosuccinic acid	4.17E-02	0.86	-1.41 (1.74)	0.78	2
1.719	Argininosuccinic acid	4.73E-02	0.86	-1.34 (1.32)	0.78	2
1.711	Argininosuccinic acid	4.72E-02	0.85	-1.28 (0.85)	0.78	2
1.756	Argininosuccinic acid	2.77E-02	0.80	-1.35 (1.11)	0.84	2
3.771	Acetylglycine*	2.73E-03	1.09	-1.51 (1.18)	0.98	1
2.068	Acetylglycine	2.14E-02	0.84	-1.49 (1.24)	0.86	1
3.766	Acetylglycine	2.05E-02	0.84	-1.22 (0.57)	0.81	1
8.461	Formic acid	2.65E-02	1.07	2.03 (1.57)	0.86	1S
3.953	Galactaric acid*	1.97E-01	1.00	-1.08 (0.24)	0.66	2
3.953	Tyrosine*	1.97E-01	1.00	-1.08 (0.24)	0.66	2
3.957	Tyrosine*	5.37E-03	0.98	-1.38 (1.01)	0.94	2
3.949	Tyrosine*	8.36E-03	0.92	-1.52 (2.33)	0.91	2
6.912	Tyrosine*	1.86E-02	0.91	-1.52 (3.6)	0.78	2
7.203	Tyrosine*	2.37E-02	0.89	-1.76 (4.81)	0.78	2
7.195	Tyrosine*	2.97E-02	0.88	-1.7 (4.03)	0.78	2
6.903	Tyrosine*	2.38E-02	0.87	-1.59 (3.47)	0.81	2
1.817	Senecioic acid	3.30E-02	0.97	1.46 (0.26)	0.80	1S
4.479	2-phosphoglycerate	3.10E-02	0.96	-1.24 (0.53)	0.84	1

4.485	2-phosphoglycerate	4.73E-02	0.77	-1.23 (0.65)	0.80	1
4.491	2-phosphoglycerate	9.82E-02	0.66	-1.47 (2.89)	0.84	1
3.897	Tagatose	7.99E-03	0.94	-1.5 (1.87)	0.94	2
3.870	Tagatose*	3.08E-02	0.83	-1.45 (2.74)	0.92	2
3.940	Creatine	8.19E-03	0.92	-1.47 (2.17)	0.89	3S
3.030	Creatine*	2.10E-02	0.85	-1.37 (1.28)	0.84	3S
3.026	Creatine*	4.60E-02	0.82	-1.44 (2.32)	0.86	3S
4.555	Ascorbic acid	2.00E-02	0.92	-1.68 (1.11)	0.84	1
4.543	Ascorbic acid	3.48E-02	0.83	-1.39 (0.96)	0.81	1
3.674	Isoleucine*	8.81E-03	0.92	-1.85 (3.27)	1.00	1
1.012	Isoleucine	4.72E-02	0.89	-1.52 (3.77)	0.75	1
1.020	Isoleucine	4.53E-02	0.89	-1.51 (3.67)	0.73	1
3.680	Isoleucine*	1.36E-02	0.89	-1.5 (3.35)	0.89	1
6.912	Hydroxyphenylacetylglycine*	1.86E-02	0.91	-1.52 (3.6)	0.78	1
7.203	Hydroxyphenylacetylglycine*	2.37E-02	0.89	-1.76 (4.81)	0.78	1
3.850	Hydroxyphenylacetylglycine	2.35E-02	0.88	-1.46 (1.18)	0.83	1
7.195	Hydroxyphenylacetylglycine*	2.97E-02	0.88	-1.7 (4.03)	0.78	1
6.903	Hydroxyphenylacetylglycine*	2.38E-02	0.87	-1.59 (3.47)	0.81	1
3.856	Hydroxyphenylacetylglycine*	2.15E-02	0.85	-1.33 (1.14)	0.81	1
4.366	Uridine*	1.00E-02	0.91	-1.38 (0.87)	0.88	3
4.241	Uridine*	1.06E-02	0.90	-1.43 (1.46)	0.88	3
3.907	Uridine*	1.49E-02	0.90	-1.46 (1.74)	0.88	3
4.139	Uridine*	1.47E-02	0.87	-1.43 (0.78)	0.88	3
3.921	Uridine*	2.22E-02	0.87	-1.45 (1.92)	0.84	3
4.229	Uridine*	2.31E-02	0.85	-1.37 (1.47)	0.88	3
4.360	Uridine*	2.21E-02	0.84	-1.4 (1.28)	0.81	3
4.355	Uridine	2.99E-02	0.83	-1.38 (1.16)	0.84	3
4.235	Uridine*	2.69E-02	0.81	-1.34 (0.92)	0.83	3
0.992	Valine	4.16E-02	0.88	-1.56 (3.68)	0.78	3
1.000	Valine	4.05E-02	0.88	-1.57 (3.3)	0.75	3
1.052	Valine	3.30E-02	0.88	-1.56 (3.6)	0.78	3
1.043	Valine	3.46E-02	0.87	-1.57 (3.5)	0.78	3
2.278	Valine	3.24E-02	0.85	-1.38 (1.7)	0.81	3
2.286	Valine	3.50E-02	0.85	-1.34 (1.34)	0.81	3
3.755	Methylimidazoleacetic acid	3.00E-02	0.87	-1.4 (1.6)	0.83	1S
3.494	Methylimidazoleacetic acid	2.68E-02	0.82	-1.45 (1.24)	0.80	1S
3.734	Glutamate	1.77E-02	0.87	-1.56 (2.04)	0.88	3
3.743	Glutamate*	2.31E-02	0.86	-1.53 (3.59)	0.91	3
3.749	Glutamate*	2.39E-02	0.86	-1.4 (1.78)	0.81	3

3.739	Glutamate*	3.20E-02	0.84	-1.48 (1.93)	0.86	3
1.608	N-methyl-a-aminoisobutyric acid	1.82E-02	0.87	-3.57 (7.89)	0.89	1S
2.141	Acetylcholine	2.72E-02	0.86	-1.64 (3.33)	0.89	1
3.743	Acetylcholine*	2.31E-02	0.86	-1.53 (3.59)	0.91	1
3.749	Acetylcholine*	2.39E-02	0.86	-1.4 (1.78)	0.81	1
3.234	Acetylcholine*	2.42E-02	0.86	-1.7 (2.33)	0.91	1
3.739	Acetylcholine*	3.20E-02	0.84	-1.48 (1.93)	0.86	1
3.866	6-phosphogluconic acid	2.09E-02	0.86	-1.43 (1.72)	0.78	2
3.870	6-phosphogluconic acid*	3.08E-02	0.83	-1.45 (2.74)	0.92	2
3.979	6-phosphogluconic acid	2.55E-02	0.81	-1.55 (2.51)	1.00	2
3.976	6-phosphogluconic acid	6.34E-02	0.73	-1.61 (8.39)	0.92	2
3.945	Phosphocreatine*	2.43E-02	0.85	-1.61 (4.21)	0.91	3S
3.030	Phosphocreatine*	2.10E-02	0.85	-1.37 (1.28)	0.84	3S
3.026	Phosphocreatine*	4.60E-02	0.82	-1.44 (2.32)	0.86	3S
3.945	Glycolic acid*	2.43E-02	0.85	-1.61 (4.21)	0.91	1S
1.254	3-Hydroxyisovaleric acid	3.22E-02	0.85	-2.08 (5.48)	0.83	1S
3.249	1,3,7-Trimethyluric acid	6.92E-02	0.82	-1.58 (2.64)	0.81	1S
3.559	Glycine	1.76E-01	0.74	-2.22 (15.2)	0.81	2S
5.922	1-Methylguanosine	1.15E-01	0.70	-1.27 (0.52)	0.84	2

Table S2. Full list of unidentified buckets for MiaPaCa-2 that were determined to be significant.

ppm	p-value	VIP	Fold Change (Error)	AUC
5.630	9.27E-04	2.54	-2.67 (0.94)	0.92
5.618	5.20E-04	2.51	-3.53 (1.3)	0.92
5.625	3.63E-06	2.45	-3.14 (0.6)	0.98
5.622	3.15E-05	2.37	-3.27 (0.84)	1.00
0.899	3.90E-02	2.18	1.18 (0.34)	0.78
0.064	7.20E-03	1.87	-14.8 (16.4)	0.88
0.167	6.25E-05	1.83	-2.17 (0.71)	1.00
1.668	1.74E-02	1.75	-1.28 (0.36)	0.84
2.889	9.78E-04	1.64	2.44 (0.88)	0.94
6.111	9.14E-01	1.57	1.01 (0.5)	0.52
2.377	8.52E-01	1.55	-1.01 (0.23)	0.50
2.015	7.89E-01	1.53	1.03 (0.62)	0.63
2.010	1.08E-01	1.48	1.19 (0.78)	0.73
6.104	9.44E-01	1.46	1.01 (0.45)	0.52
3.416	1.98E-01	1.46	1.22 (0.22)	0.69
5.406	6.19E-01	1.45	-1.09 (0.74)	0.59
5.397	4.84E-01	1.42	-1.12 (0.45)	0.63

5.998	5.05E-03	1.38	-1.29 (0.19)	0.88
5.562	4.18E-01	1.37	-1.12 (0.46)	0.58
3.202	7.01E-01	1.36	1.04 (0.51)	0.52
8.150	5.98E-01	1.36	1.59 (8.49)	0.52
3.574	5.86E-01	1.36	-1.08 (0.58)	0.58
2.674	9.70E-01	1.34	1 (0.16)	0.53
1.555	8.66E-01	1.31	-1.02 (0.5)	0.55
7.551	6.51E-01	1.29	-1.19 (2.68)	0.72
1.994	1.46E-01	1.29	1.11 (0.32)	0.70
5.377	5.92E-01	1.26	-1.15 (0.67)	0.55
1.986	7.48E-01	1.25	1.02 (0.28)	0.58
5.410	8.79E-01	1.25	1.05 (1.31)	0.50
8.220	9.12E-01	1.24	1.05 (3.26)	0.59
1.284	4.49E-01	1.24	-1.22 (3.33)	0.55
2.341	4.45E-01	1.23	1.05 (0.18)	0.61
2.332	6.94E-01	1.23	1.03 (0.14)	0.53
3.264	1.18E-01	1.22	1.21 (0.43)	0.75
2.359	9.81E-01	1.22	1 (0.17)	0.50
1.467	5.10E-01	1.22	-1.1 (1.09)	0.55
3.209	2.82E-01	1.20	1.08 (0.17)	0.63
8.005	6.56E-01	1.19	-1.41 (6.67)	0.58
2.109	4.50E-02	1.19	-1.14 (0.24)	0.78
1.336	3.74E-01	1.19	1.11 (0.82)	0.73

5.372	4.83E-03	1.19	-1.82<br>(0.69)	0.97
2.925	2.14E-01	1.19	1.27<br>(0.29)	0.67
5.803	4.43E-01	1.19	1.53<br>(6.07)	0.53
3.532	1.04E-01	1.18	1.26<br>(1.12)	0.66
2.669	1.37E-01	1.17	1.16<br>(0.36)	0.70
1.278	4.43E-01	1.17	-1.17<br>(2.18)	0.53
3.614	1.50E-01	1.16	1.15<br>(0.55)	0.73
1.327	4.99E-01	1.16	1.08<br>(0.74)	0.67
2.322	3.94E-01	1.16	1.06<br>(0.19)	0.59
3.791	6.78E-01	1.16	-1.03<br>(0.17)	0.53
3.047	5.31E-01	1.16	1.04<br>(0.35)	0.58
3.039	2.48E-01	1.15	1.08<br>(0.32)	0.69
1.250	2.86E-01	1.13	-1.22<br>(2.13)	0.56
1.650	3.05E-01	1.12	1.13<br>(0.098)	0.78
2.367	9.05E-01	1.12	-1.01<br>(0.18)	0.52
5.558	1.02E-02	1.12	-1.37<br>(0.38)	0.88
2.695	4.77E-01	1.12	1.06<br>(0.19)	0.55
2.990	9.88E-01	1.11	1<br>(0.34)	0.50
2.093	5.72E-03	1.11	-1.27<br>(0.63)	0.91
4.120	4.04E-01	1.10	1.05<br>(0.17)	0.63
8.198	9.52E-01	1.10	1.01<br>(0.47)	0.52
4.021	8.28E-03	1.10	-1.37<br>(0.39)	0.91
3.544	7.80E-02	1.09	1.32<br>(1.42)	0.69

3.626	9.96E-02	1.08	1.23 (1.2)	0.66
8.840	6.69E-02	1.08	0.21 (11.1)	0.77
3.638	1.17E-01	1.07	1.23 (1.69)	0.64
3.933	5.49E-01	1.05	-1.03 (0.25)	0.53
3.225	5.11E-01	1.05	-1.28 (1.21)	0.53
4.109	1.94E-01	1.05	1.1 (0.48)	0.66
2.030	6.79E-01	1.04	-1.02 (0.27)	0.55
1.259	1.97E-01	1.04	-1.28 (2.97)	0.59
5.812	5.96E-01	1.04	1.27 (3.98)	0.56
1.266	1.77E-01	1.04	-1.37 (4.12)	0.59
3.477	7.47E-01	1.04	-1.08 (0.78)	0.58
2.662	7.94E-01	1.03	1.06 (1.1)	0.66
2.123	1.20E-01	1.03	-1.13 (0.32)	0.72
1.275	1.48E-01	1.02	-1.4 (3.63)	0.59
2.704	2.17E-01	1.02	-1.1 (0.21)	0.67
2.407	3.62E-01	1.02	-1.13 (0.39)	0.66
1.461	3.10E-01	1.02	-1.19 (1.43)	0.58
3.285	7.90E-02	1.01	1.33 (1.79)	0.72
3.296	1.05E-01	1.01	1.25 (1.33)	0.70
2.590	9.82E-01	1.01	-1 (0.19)	0.50
1.273	2.17E-01	1.00	-1.34 (2.51)	0.67
1.901	2.07E-01	1.00	-1.2 (1.05)	0.59
5.553	1.04E-02	1.00	-1.44 (0.69)	0.86

5.549	8.79E-03	0.99	-1.52 (0.67)	0.95
3.273	5.39E-02	0.99	1.35 (1.31)	0.75
3.608	1.01E-01	0.98	-1.3 (0.75)	0.72
3.537	4.95E-02	0.96	1.45 (1.86)	0.70
2.666	7.24E-01	0.96	1.08 (1.26)	0.73
2.042	1.36E-01	0.95	-1.14 (0.49)	0.72
2.058	2.04E-01	0.94	-1.15 (0.49)	0.73
7.373	3.57E-02	0.94	-1.39 (2.14)	0.77
1.111	8.96E-03	0.92	-1.79 (1.41)	0.88
2.399	1.15E-01	0.92	-1.19 (0.67)	0.70
2.133	1.77E-01	0.91	-1.12 (0.38)	0.70
7.339	2.67E-02	0.90	-1.66 (4.68)	0.78
3.795	4.76E-02	0.90	-1.17 (0.61)	0.80
2.023	7.59E-02	0.90	-1.17 (0.57)	0.73
7.432	2.20E-02	0.90	-1.74 (4.01)	0.78
4.567	2.86E-02	0.89	-1.52 (0.82)	0.84
3.881	1.22E-02	0.89	-1.61 (1.97)	0.88
0.957	5.18E-02	0.89	-1.52 (3.12)	0.73
1.750	5.12E-02	0.89	-1.43 (1.98)	0.77
1.681	6.57E-02	0.89	-1.27 (1.13)	0.75
3.811	1.97E-02	0.88	-1.38 (1.26)	0.75
4.263	2.46E-02	0.88	-1.54 (2.02)	0.75
0.964	4.82E-02	0.88	-1.56 (3.47)	0.75

7.424	2.45E-02	0.88	-1.69 (4.13)	0.78
0.975	4.33E-02	0.88	-1.54 (2.94)	0.77
0.968	4.45E-02	0.87	-1.52 (2.86)	0.77
2.291	5.06E-02	0.87	-1.25 (0.93)	0.75
4.191	2.13E-02	0.87	-1.41 (1.29)	0.78
7.330	3.27E-02	0.87	-1.7 (3.23)	0.77
1.744	2.04E-02	0.87	-1.48 (1.82)	0.89
3.021	7.50E-02	0.86	-1.35 (1.44)	0.73
3.835	4.56E-02	0.86	-1.49 (1.89)	0.77
3.884	2.15E-02	0.86	-1.48 (1.69)	0.95
1.481	2.91E-02	0.86	1.34 (1.03)	0.78
2.282	4.94E-02	0.86	-1.35 (1.51)	0.70
3.504	7.68E-02	0.86	-1.59 (2.87)	0.75
2.210	5.20E-02	0.86	-1.32 (1.43)	0.72
2.294	5.87E-02	0.85	-1.24 (0.87)	0.77
3.436	3.95E-02	0.85	1.5 (1.09)	0.78
3.762	3.63E-02	0.85	-1.31 (0.8)	0.80
1.736	2.42E-02	0.85	-1.43 (1.43)	0.91
3.429	9.72E-02	0.85	1.36 (0.84)	0.72
2.648	4.49E-02	0.85	-1.73 (2.55)	0.75
2.195	4.90E-02	0.85	-1.31 (0.97)	0.78
2.268	4.69E-02	0.85	-1.29 (1.07)	0.75
2.274	3.85E-02	0.85	-1.33 (1.2)	0.75

8.830	1.15E-01	0.84	6.33 (14.7)	0.70
2.639	5.90E-02	0.84	-1.58 (2.19)	0.72
2.395	7.66E-02	0.84	-1.3 (0.99)	0.70
4.648	4.71E-02	0.84	-1.78 (1.62)	0.75
1.489	3.83E-02	0.84	1.32 (1.06)	0.75
2.657	3.50E-02	0.84	-1.54 (1.75)	0.78
3.650	1.97E-02	0.84	-1.38 (0.69)	0.83
4.657	6.50E-02	0.83	-1.64 (1.36)	0.73
3.481	5.04E-02	0.83	-1.3 (1.83)	0.73
2.084	2.62E-02	0.83	-1.54 (1.06)	0.83
5.975	2.75E-02	0.82	-6.06 (5.13)	0.80
5.368	2.28E-02	0.82	-1.49 (0.65)	0.89
7.877	1.27E-01	0.82	-1.36 (0.93)	0.75
3.241	5.55E-02	0.80	-1.53 (1.33)	0.80
3.257	8.40E-02	0.78	-1.45 (2.57)	0.75
2.080	5.54E-02	0.78	-1.14 (0.34)	0.73
5.213	1.01E-01	0.77	0.32 (20.2)	0.70
5.363	4.90E-02	0.77	-1.39 (0.51)	0.86
5.209	9.70E-02	0.77	0.19 (30)	0.70
2.415	9.21E-02	0.77	-1.21 (0.51)	0.72
5.358	4.65E-02	0.76	-1.49 (0.73)	0.88
3.619	1.30E-01	0.76	-1.18 (0.99)	0.75
1.936	8.25E-02	0.75	-1.2 (0.76)	0.73

2.073	5.90E-02	0.75	-1.26 (0.45)	0.83
3.062	7.72E-02	0.74	-1.39 (1.77)	0.73
3.135	1.28E-01	0.74	-1.45 (1.93)	0.70
7.886	7.06E-02	0.73	-1.32 (0.65)	0.78
6.098	1.14E-01	0.72	1.48 (4.05)	0.77
3.359	7.55E-02	0.72	-3.24 (3.75)	0.86
1.947	1.14E-01	0.70	-1.16 (0.43)	0.73
1.319	1.22E-01	0.69	-1.47 (1.21)	0.70
3.595	1.36E-01	0.65	-1.14 (0.39)	0.72
5.928	5.33E-01	0.64	-1.1 (0.55)	0.73
4.432	1.29E-01	0.58	-1.18 (0.62)	0.72

Table S3. Identified metabolites whose abundance were significantly different depending on drying technique in the Panc-1 cell line.

ppm	Metabolite Identification	p-value	VIP	Fold Change (Error)	AUC	Rank
3.706	Dehydroascorbic acid	5.61E-12	2.00	-2.4 (0.26)	1.00	2
3.713	Dehydroascorbic acid	1.50E-07	1.85	-1.51 (0.17)	1.00	2
3.828	Dehydroascorbic acid*	8.35E-04	1.32	-1.14 (0.14)	0.93	2
4.291	Dehydroascorbic acid*	4.80E-03	1.23	-1.16 (0.19)	0.84	2
3.784	Dehydroascorbic acid*	1.36E-02	1.16	-1.16 (0.24)	0.82	2
3.719	Dehydroascorbic acid	4.02E-02	1.06	-1.13 (0.14)	0.80	2
1.921	Acetate	2.89E-08	1.99	2.58 (0.14)	1.00	1S
3.391	1,3,7-trimethyluric acid	1.34E-12	1.95	-3.3 (0.34)	1.00	1S
3.355	1,3,7-trimethyluric acid	3.79E-04	1.66	2.53 (0.064)	0.83	1S
3.226	1,3,7-trimethyluric acid	1.48E-02	1.07	2.89 (0.084)	0.82	1S
2.885	Trimethylamine	1.50E-05	1.81	1.85 (0.4)	0.99	1S
3.813	2-amino-3-phosphonopropionic acid*	1.57E-06	1.68	-1.35 (0.19)	0.97	2
2.086	2-amino-3-phosphonopropionic acid	4.33E-04	1.37	-1.56 (0.41)	0.93	2
3.828	2-amino-3-phosphonopropionic acid*	8.35E-04	1.32	-1.14 (0.14)	0.93	2
1.703	2-amino-3-phosphonopropionic acid	2.24E-03	1.25	-1.13 (0.11)	0.90	2
3.813	Alanine*	1.57E-06	1.68	-1.35 (0.19)	0.97	3
3.802	Alanine	2.45E-06	1.65	-1.43 (0.25)	0.99	3
1.844	N-acetylneuraminic acid	4.96E-04	1.59	1.28 (0.18)	0.94	1
1.930	N-acetylneuraminic acid	7.89E-02	1.11	1.06 (0.062)	0.69	1
2.059	N-acetylneuraminic acid	1.14E-01	0.98	1.07 (0.25)	0.71	1
3.046	Phosphocreatine	8.31E-06	1.58	-1.81 (0.62)	1.00	3S
3.946	Phosphocreatine	9.78E-03	1.11	-1.14 (0.16)	0.84	3S
3.695	Isoleucine	1.94E-05	1.57	-1.26 (0.2)	1.00	2
1.253	Isoleucine	5.15E-05	1.50	-1.52 (0.4)	0.98	2
1.261	Isoleucine	1.49E-03	1.32	-1.22 (0.14)	0.89	2
1.268	Isoleucine	7.21E-03	1.23	-1.17 (0.17)	0.86	2
1.277	Isoleucine	6.09E-03	1.21	-1.18 (0.14)	0.86	2
1.247	Isoleucine	6.25E-03	1.18	-1.45 (0.31)	0.89	2
2.000	Isoleucine	4.73E-03	1.16	-1.17 (0.15)	0.87	2
0.944	Isoleucine	2.11E-02	1.14	-1.13 (0.25)	0.81	2
0.934	Isoleucine	2.89E-02	1.08	-1.11 (0.22)	0.82	2
1.020	Isoleucine	4.26E-02	1.06	-1.14 (0.27)	0.80	2
1.011	Isoleucine	5.61E-02	1.05	-1.13 (0.3)	0.78	2
0.952	Isoleucine	4.07E-02	1.04	-1.11 (0.2)	0.79	2

1.468	Isoleucine*	5.69E-02	0.84	-1.09 (0.12)	0.77	2
8.482	NAD	1.58E-04	1.52	-2.9 (0.99)	0.94	1
9.343	NAD	8.55E-04	1.31	-1.29 (0.21)	0.91	1
9.148	NAD	3.11E-03	1.26	-1.24 (0.27)	0.88	1
9.139	NAD	3.99E-03	1.19	-1.23 (0.23)	0.88	1
6.043	NAD*	1.13E-02	1.14	-1.32 (0.25)	0.84	1
6.099	NAD*	6.58E-03	1.14	-1.24 (0.22)	0.84	1
6.093	NAD*	1.32E-02	1.09	-1.27 (0.27)	0.90	1
3.955	Glycolic acid*	5.34E-05	1.49	-1.34 (0.22)	0.98	1S
3.955	Serine*	5.34E-05	1.49	-1.34 (0.22)	0.98	2
3.965	Serine	1.93E-02	1.01	-1.14 (0.2)	0.82	2
3.955	Tyrosine*	5.34E-05	1.49	-1.34 (0.22)	0.98	1
6.913	Tyrosine*	5.05E-02	1.02	-1.1 (0.15)	0.77	1
6.901	Tyrosine*	1.30E-01	0.95	-1.09 (0.2)	0.71	1
1.912	Lysine*	2.32E-03	1.44	1.17 (0.078)	0.93	2
3.020	Lysine	5.17E-03	1.18	-1.15 (0.1)	0.89	2
1.711	Lysine*	8.35E-03	1.12	-1.12 (0.12)	0.86	2
3.029	Lysine	1.61E-02	1.07	-1.13 (0.15)	0.82	2
1.718	Lysine*	3.91E-02	0.97	-1.09 (0.13)	0.77	2
1.727	Lysine*	1.00E-01	0.90	-1.06 (0.11)	0.71	2
1.738	Lysine*	1.15E-01	0.85	-1.07 (0.13)	0.71	2
1.468	Lysine*	5.69E-02	0.84	-1.09 (0.12)	0.77	2
1.912	Argininosuccinic acid*	2.32E-03	1.44	1.17 (0.078)	0.93	2
1.696	Argininosuccinic acid	1.24E-03	1.31	-1.14 (0.1)	0.92	2
4.256	Argininosuccinic acid*	3.48E-03	1.25	-1.39 (0.35)	0.89	2
1.691	Argininosuccinic acid	5.96E-03	1.14	-1.16 (0.19)	0.84	2
1.711	Argininosuccinic acid*	8.35E-03	1.12	-1.12 (0.12)	0.86	2
1.686	Argininosuccinic acid	2.99E-02	0.97	-1.09 (0.1)	0.80	2
1.718	Argininosuccinic acid*	3.91E-02	0.97	-1.09 (0.13)	0.77	2
1.727	Argininosuccinic acid*	1.00E-01	0.90	-1.06 (0.11)	0.71	2
1.738	Argininosuccinic acid*	1.15E-01	0.85	-1.07 (0.13)	0.71	2
3.868	Tagatose	1.91E-04	1.44	-1.22 (0.11)	0.93	2
4.052	Xylulose	2.02E-04	1.42	-1.28 (0.13)	0.94	2
4.046	Xylulose	5.89E-04	1.40	-1.23 (0.17)	0.92	2
3.574	Xylulose	3.12E-01	1.07	-1.05 (0.2)	0.62	2
4.364	Xylulose	2.69E-02	1.03	-1.19 (0.22)	0.79	2
4.214	Phosphoserine	2.06E-04	1.42	-1.28 (0.25)	0.96	2
4.000	Phosphoserine	2.62E-02	1.00	-1.12 (0.2)	0.81	2
8.352	Adenosine	1.57E-01	1.40	1.1 (0.19)	0.83	2
6.111	Adenosine	3.00E-01	1.10	1.06 (0.15)	0.63	2
4.425	Adenosine	4.67E-02	0.87	-1.18 (0.21)	0.79	2

2.291	Valine	3.00E-04	1.39	-1.14 (0.076)	0.96	3
2.260	Valine*	1.79E-03	1.36	-1.16 (0.094)	0.91	3
2.269	Valine	8.69E-04	1.31	-1.14 (0.094)	0.97	3
2.299	Valine	2.67E-03	1.24	-1.11 (0.052)	0.86	3
2.284	Valine	8.08E-03	1.15	-1.12 (0.12)	0.83	3
1.001	Valine	5.65E-02	1.09	-1.11 (0.22)	0.78	3
2.265	Valine	1.18E-02	1.07	-1.09 (0.093)	0.81	3
1.051	Valine	6.47E-02	1.06	-1.11 (0.22)	0.77	3
1.042	Valine	6.86E-02	1.03	-1.1 (0.23)	0.78	3
0.991	Valine	1.00E-01	1.02	-1.09 (0.24)	0.74	3
2.275	Valine	3.24E-02	1.02	-1.09 (0.12)	0.76	3
2.260	Ribose*	1.79E-03	1.36	-1.16 (0.094)	0.91	2
2.972	Glutathione	5.88E-04	1.36	-1.37 (0.22)	0.91	3
2.983	Glutathione	4.92E-04	1.35	-1.24 (0.15)	0.96	3
2.940	Glutathione	6.16E-04	1.35	-1.35 (0.22)	0.90	3
2.579	Glutathione	6.19E-04	1.35	-1.28 (0.22)	0.92	3
2.948	Glutathione	1.02E-03	1.32	-1.34 (0.23)	0.91	3
2.965	Glutathione	1.07E-03	1.31	-1.34 (0.24)	0.93	3
2.588	Glutathione	1.29E-03	1.29	-1.26 (0.29)	0.91	3
2.570	Glutathione	1.80E-03	1.28	-1.25 (0.24)	0.88	3
2.553	Glutathione	2.05E-03	1.28	-1.25 (0.28)	0.90	3
2.931	Glutathione	2.59E-03	1.25	-1.35 (0.27)	0.88	3
2.988	Glutathione	2.96E-03	1.24	-1.2 (0.11)	0.89	3
2.186	Glutathione	6.94E-03	1.18	-1.17 (0.21)	0.82	3
2.561	Glutathione	8.42E-03	1.18	-1.19 (0.26)	0.84	3
3.784	Glutathione*	1.36E-02	1.16	-1.16 (0.24)	0.82	3
2.177	Glutathione	1.21E-02	1.14	-1.21 (0.33)	0.83	3
2.160	Glutathione	1.29E-02	1.11	-1.14 (0.16)	0.80	3
2.167	Glutathione	2.86E-02	1.05	-1.17 (0.33)	0.78	3
2.977	Glutathione	2.07E-02	1.04	-1.17 (0.16)	0.79	3
2.534	Glutathione	2.40E-02	1.02	-1.11 (0.19)	0.76	3
2.545	Glutathione	4.57E-02	1.00	-1.13 (0.3)	0.76	3
2.674	Glutathione	5.29E-02	0.87	1.21 (0.14)	0.77	3
8.175	Hypoxanthine	6.56E-04	1.35	-1.32 (0.22)	0.91	1S
8.429	1-Methyladenosine	7.62E-04	1.32	-1.3 (0.24)	0.91	1
4.491	1-Methyladenosine	2.65E-03	1.32	-1.48 (0.33)	0.91	1
4.291	1-Methyladenosine*	4.80E-03	1.23	-1.16 (0.19)	0.84	1
6.043	1-Methyladenosine*	1.13E-02	1.14	-1.32 (0.25)	0.84	1
6.099	1-Methyladenosine*	6.58E-03	1.14	-1.24 (0.22)	0.84	1
8.276	1-Methyladenosine	2.03E-02	1.13	-1.42 (0.57)	0.84	1
6.093	1-Methyladenosine*	1.32E-02	1.09	-1.27 (0.27)	0.90	1
3.828	Mannose*	8.35E-04	1.32	-1.14 (0.14)	0.93	2

3.673	Mannose	3.43E-02	1.07	-1.11 (0.26)	0.81	2
3.822	Mannose	2.21E-02	1.02	-1.07 (0.11)	0.79	2
1.400	Lactic acid	2.89E-03	1.32	-1.14 (0.12)	0.87	3
1.408	Lactic acid	5.60E-03	1.23	-1.12 (0.12)	0.89	3
3.210	Acetylcholine*	1.38E-02	1.28	1.19 (0.17)	0.82	1
2.154	Acetylcholine	1.39E-02	1.12	1.18 (0.08)	0.83	1
3.210	Glycerophosphocholine*	1.38E-02	1.28	1.19 (0.17)	0.82	3
4.332	Glycerophosphocholine	5.52E-02	0.85	-1.09 (0.18)	0.77	3
5.987	Cytosine	1.49E-03	1.28	-2.12 (0.93)	0.90	1
5.979	Cytosine	3.06E-02	0.95	-1.99 (1.59)	0.77	1
4.256	Threonine*	3.48E-03	1.25	-1.39 (0.35)	0.89	3
3.591	Threonine	8.51E-02	1.05	-1.11 (0.29)	0.73	3
1.338	Threonine	2.26E-02	1.03	-1.14 (0.16)	0.80	3
2.407	Oxalacetic acid	4.01E-03	1.23	-1.17 (0.18)	0.89	1S
7.957	6-Dimethylaminopurine	3.64E-03	1.20	-1.77 (1.09)	0.89	1S
2.804	Aspartic acid	8.81E-03	1.17	1.29 (0.087)	0.87	2
3.249	Glucose	1.90E-02	1.14	1.09 (0.11)	0.83	2
7.552	Uracil	1.24E-02	1.07	1.31 (0.18)	0.81	1
7.543	Uracil	1.47E-02	1.06	1.29 (0.3)	0.84	1
1.609	N-methyl-a-aminoisobutyric acid	1.33E-02	1.05	-2.52 (5.53)	0.92	1S
6.913	Hydroxyphenylacetylglycine*	5.05E-02	1.02	-1.1 (0.15)	0.77	1
6.901	Hydroxyphenylacetylglycine*	1.30E-01	0.95	-1.09 (0.2)	0.71	1
3.934	Creatine	1.57E-01	1.01	-1.07 (0.19)	0.72	3S
1.378	Mevalonic acid	3.52E-02	0.95	1.38 (0.28)	0.78	1
4.058	Creatinine	4.80E-02	0.91	-1.19 (0.22)	0.77	1S

*** indicates that these buckets occurred in an overlapped part of the spectrum**

Table S4. Full list of unidentified buckets for Panc-1 that were determined to be significant.

ppm	p-value	VIP	Fold Change (Error)	AUC
2.008	1.12E-08	1.86	-2.15 (0.59)	1.00
0.058	8.08E-04	1.68	-13.2 (10.7)	0.90
0.168	5.85E-05	1.66	-1.44 (0.51)	1.00
3.084	6.51E-06	1.60	-1.71 (0.33)	0.99
2.397	1.32E-05	1.58	-1.15 (0.074)	0.98
3.101	1.52E-05	1.56	-1.37 (0.26)	0.98
6.942	1.87E-04	1.55	-2.99 (0.81)	0.91
1.110	1.30E-04	1.47	-1.5 (0.69)	1.00
2.416	1.07E-04	1.45	-1.12 (0.059)	0.93
1.099	5.38E-04	1.42	-1.33 (0.16)	0.92
2.035	5.44E-04	1.36	-1.15 (0.1)	0.91
4.299	5.71E-04	1.35	-1.32 (0.28)	0.92
4.383	1.12E-03	1.31	-1.34 (0.31)	0.90
5.993	1.23E-03	1.31	-1.94 (0.56)	0.88
2.029	1.60E-03	1.30	-1.15 (0.12)	0.90
3.574	2.48E-01	1.24	1.1 (0.22)	0.67
2.043	3.37E-03	1.21	-1.12 (0.16)	0.87

5.622	6.97E-03	1.20	-1.32 (0.19)	0.88
2.209	5.11E-03	1.16	-1.07 (0.062)	0.82
3.398	4.91E-02	1.12	-1.27 (0.17)	0.77
3.069	7.41E-01	1.11	1.02 (0.34)	0.66
8.193	7.77E-02	1.09	-1.17 (0.31)	0.79
2.202	1.13E-02	1.07	-1.09 (0.12)	0.82
8.461	1.95E-02	1.07	3.16 (0.15)	0.74
4.282	8.12E-01	1.06	-1.01 (0.2)	0.50
4.287	6.97E-01	1.06	-1.02 (0.21)	0.53
5.615	1.93E-02	1.06	-1.36 (0.36)	0.81
1.155	4.77E-01	1.05	1.2 (0.7)	0.59
0.976	7.81E-02	1.05	-1.09 (0.23)	0.70
4.445	7.71E-01	1.05	1.02 (0.23)	0.57
2.825	2.16E-02	1.04	1.19 (0.11)	0.76
0.958	7.89E-02	1.03	-1.09 (0.22)	0.73
3.286	1.47E-01	1.03	-1.08 (0.27)	0.69
0.966	9.89E-02	1.03	-1.09 (0.23)	0.69
0.899	1.76E-02	1.02	-1.12 (0.14)	0.78
2.129	4.50E-02	1.02	1.15 (0.1)	0.73
3.189	6.78E-01	1.02	-1.03 (0.27)	0.56
8.246	8.54E-01	1.02	-1.01 (0.096)	0.53
5.811	2.24E-02	1.02	1.49 (0.39)	0.78

7.432	5.84E-02	1.02	-1.13 (0.21)	0.76
3.126	1.20E-01	1.02	-1.09 (0.26)	0.72
3.273	2.18E-01	1.02	-1.07 (0.27)	0.69
3.296	2.75E-01	1.02	-1.06 (0.23)	0.63
3.155	2.58E-01	1.01	-1.07 (0.23)	0.67
7.887	5.74E-01	1.01	1.04 (0.22)	0.51
7.339	6.86E-02	1.01	-1.12 (0.22)	0.74
3.638	8.62E-02	1.01	-1.1 (0.33)	0.74
3.680	4.23E-02	1.01	-1.09 (0.21)	0.76
5.522	3.11E-02	1.01	-3.02 (2.08)	0.72
3.535	2.53E-01	1.01	-1.07 (0.29)	0.67
2.545	4.57E-02	1.00	-1.13 (0.3)	0.76
3.053	1.57E-01	1.00	-1.08 (0.5)	0.71
3.547	1.83E-01	0.99	-1.08 (0.31)	0.70
5.968	3.21E-02	0.99	-1.82 (0.77)	0.78
2.120	4.36E-02	0.99	1.13 (0.084)	0.77
7.381	4.69E-02	0.98	-1.1 (0.13)	0.74
5.628	3.41E-02	0.97	-1.29 (0.26)	0.81
4.236	9.68E-02	0.97	-1.09 (0.27)	0.72
2.350	6.84E-02	0.97	1.18 (0.15)	0.71
5.803	4.05E-02	0.97	1.38 (0.4)	0.73
7.423	1.14E-01	0.97	-1.11 (0.23)	0.73

1.681	4.30E-02	0.97	-1.08 (0.11)	0.73
2.340	6.27E-02	0.97	1.15 (0.11)	0.73
5.366	4.83E-02	0.97	1.49 (0.51)	0.76
3.145	1.62E-01	0.97	-1.09 (0.17)	0.71
2.367	8.74E-02	0.97	1.12 (0.12)	0.73
7.329	1.90E-01	0.96	-1.1 (0.27)	0.72
8.217	9.02E-02	0.96	1.2 (0.35)	0.72
2.597	5.73E-02	0.95	-1.17 (0.24)	0.73
5.902	7.27E-02	0.93	1.35 (0.44)	0.73
2.701	3.79E-02	0.92	-1.5 (1.28)	0.81
3.236	1.32E-01	0.92	-1.09 (0.27)	0.71
3.877	4.83E-02	0.92	-1.13 (0.25)	0.70
5.189	6.35E-02	0.92	1.7 (0.75)	0.71
3.173	1.04E-01	0.91	-1.13 (0.18)	0.76
1.328	4.47E-02	0.90	-1.11 (0.15)	0.72
3.426	1.10E-01	0.90	-1.1 (0.27)	0.70
3.432	7.59E-02	0.90	-1.1 (0.26)	0.77
2.101	4.08E-02	0.89	1.22 (0.13)	0.79
5.998	6.37E-02	0.88	-1.54 (0.72)	0.74
3.417	6.46E-02	0.87	-1.09 (0.16)	0.73
2.015	6.88E-02	0.85	-1.09 (0.13)	0.74
2.476	7.74E-02	0.84	1.26 (0.45)	0.74

1.132	1.87E-01	0.84	-1.16 (0.35)	0.71
3.688	1.07E-01	0.83	-1.08 (0.24)	0.70
5.211	1.35E-01	0.76	1.46 (3.97)	0.72
2.525	1.98E-01	0.74	-1.05 (0.084)	0.71
5.928	1.01E-01	0.74	1.65 (0.92)	0.79
4.513	1.18E-01	0.72	-1.23 (0.33)	0.79
1.676	1.40E-01	0.70	-1.05 (0.11)	0.70
1.977	1.38E-01	0.69	-1.1 (0.18)	0.70
2.050	2.46E-01	0.68	-1.05 (0.13)	0.71
2.216	1.46E-01	0.67	-1.05 (0.11)	0.71
4.483	1.45E-01	0.67	-1.15 (0.24)	0.77
3.257	1.65E-01	0.66	1.07 (0.099)	0.70
2.439	1.60E-01	0.65	1.09 (0.14)	0.71
5.913	3.19E-01	0.61	1.15 (0.29)	0.73
4.569	2.10E-01	0.59	-1.2 (0.39)	0.70
1.312	2.05E-01	0.59	-1.26 (0.29)	0.72
5.922	4.13E-01	0.54	1.16 (0.36)	0.71
4.544	3.00E-01	0.49	-1.17 (0.32)	0.76
4.477	4.06E-01	0.40	-1.1 (0.25)	0.72

Table S5. Identified metabolites that were found significant for AsPC-1 cell line. Only first significant buckets for each metabolite are shown.

ppm	Metabolite Identification	p-value	VIP	Fold Change (Error)	AUC	Rank
1.920	Acetate	4.14E-15	2.28	3.31 (0.16)	1.00	2S
3.391	1,3,7-trimethyluric acid	3.75E-08	2.11	-2.02 (0.37)	1.00	1S
3.359	1,3,7-trimethyluric acid	4.70E-06	1.96	-5.46 (5.51)	1.00	1S
3.353	1,3,7-trimethyluric acid	1.90E-04	1.74	-1.46 (0.71)	0.91	1S
1.187	3-hydroxybutyrate	6.62E-08	2.10	-2.62 (0.67)	1.00	1
2.391	Malic acid	3.24E-07	2.06	-2.16 (1.08)	1.00	1
2.703	Malic acid	3.83E-04	1.69	-1.44 (0.46)	0.91	1
2.663	Malic acid	2.65E-03	1.50	-1.32 (0.22)	0.89	1
2.736	Sarcosine	7.37E-07	2.03	-3.56 (1.4)	1.00	1S
6.099	1-methyladenosine*	3.07E-06	1.97	2.5 (0.57)	0.99	1
6.093	1-methyladenosine*	3.27E-05	1.86	3.02 (1.15)	1.00	1
6.037	1-methyladenosine*	3.88E-05	1.85	5.91 (2.28)	0.97	1
6.044	1-methyladenosine*	1.83E-04	1.74	2.38 (0.67)	0.93	1
8.177	1-methyladenosine	2.22E-04	1.73	2.05 (0.35)	0.97	1
8.430	1-methyladenosine	1.60E-03	1.55	1.41 (0.16)	0.90	1
8.461	1-methyladenosine	9.53E-03	1.33	1.25 (0.39)	0.83	1
6.099	Inosine*	3.07E-06	1.97	2.5 (0.57)	0.99	2
6.093	Inosine*	3.27E-05	1.86	3.02 (1.15)	1.00	2
3.481	Glucose*	3.68E-06	1.97	1.5 (0.31)	0.99	1
5.237	Glucose*	1.38E-03	1.57	1.54 (0.33)	0.91	1
4.648	Glucose*	1.89E-03	1.54	-1.49 (0.96)	0.92	1
4.658	Glucose*	2.29E-03	1.51	-1.48 (0.98)	0.94	1
5.244	Glucose*	2.47E-03	1.51	1.46 (0.46)	0.84	1
3.829	Glucose*	2.93E-03	1.49	1.24 (0.29)	0.87	1
3.714	Glucose*	3.87E-02	1.10	1.15 (0.21)	0.81	1
3.481	1-methylguanine	3.68E-06	1.97	1.5 (0.31)	0.99	2S
1.608	N-methyl-a-aminoisobutyric acid	4.44E-06	1.96	-5.49 (2.75)	0.99	1S
4.189	Phosphocholine	8.65E-06	1.93	-1.54 (0.51)	1.00	1
3.209	Acetylcarnitine	1.39E-05	1.90	1.52 (0.22)	0.98	1
4.522	Ascorbic acid	2.33E-05	1.88	-2.01 (0.91)	1.00	2
6.037	NAD*	3.88E-05	1.85	5.91 (2.28)	0.97	1
6.044	NAD*	1.83E-04	1.74	2.38 (0.67)	0.93	1
9.343	NAD	8.83E-04	1.61	1.56 (0.21)	0.93	1
2.226	3-Cresotinic acid	5.11E-05	1.83	-1.65 (0.99)	1.00	1
5.998	Cytidine Triphosphate	1.00E-04	1.79	1.97 (1.22)	0.97	2

5.993	Cytidine Triphosphate	2.86E-04	1.71	1.75 (0.8)	0.92	2
3.803	Guanidoacetic acid	1.47E-04	1.76	-1.67 (2.02)	1.00	1S
4.169	Glyceric acid	2.02E-04	1.74	-1.36 (0.41)	1.00	2
4.256	Threonine*	2.75E-04	1.71	-1.41 (0.31)	0.98	2
1.337	Threonine*	3.97E-02	1.10	1.13 (0.27)	0.77	2
4.256	Argininosuccinic acid*	2.75E-04	1.71	-1.41 (0.31)	0.98	2
2.545	Argininosuccinic acid*	2.49E-03	1.51	-1.49 (0.45)	0.92	2
1.930	Argininosuccinic acid*	2.62E-03	1.50	1.24 (0.23)	0.84	2
2.535	Argininosuccinic acid*	8.23E-03	1.36	-1.39 (0.21)	0.86	2
2.570	Argininosuccinic acid	9.13E-03	1.34	-1.32 (0.43)	0.82	2
2.553	Argininosuccinic acid*	1.57E-02	1.26	-1.31 (0.31)	0.82	2
1.690	Argininosuccinic acid*	3.04E-02	1.15	-1.17 (0.56)	0.81	2
1.697	Argininosuccinic acid*	3.49E-02	1.12	-1.17 (0.52)	0.77	2
1.377	Mevalonic acid	3.04E-04	1.71	1.57 (0.17)	0.92	2
1.380	Mevalonic acid	7.93E-03	1.36	1.38 (0.13)	0.80	2
2.495	3-hydroxy-3-methylglutarate	6.61E-04	1.64	1.51 (0.17)	0.90	1
2.008	Acetylglycine	8.94E-04	1.61	-1.29 (0.28)	0.88	1
4.360	Hydroxyacetone	1.00E-03	1.60	-1.38 (0.77)	0.93	1S
2.169	Hydroxyacetone	5.87E-03	1.40	-1.31 (0.39)	0.87	1S
5.237	Glucuronate*	1.38E-03	1.57	1.54 (0.33)	0.91	2
4.648	Glucuronate*	1.89E-03	1.54	-1.49 (0.96)	0.92	2
4.658	Glucuronate*	2.29E-03	1.51	-1.48 (0.98)	0.94	2
5.244	Glucuronate*	2.47E-03	1.51	1.46 (0.46)	0.84	2
3.557	Glucuronate	6.69E-03	1.38	1.36 (0.36)	0.87	2
3.571	Glucuronate	7.21E-03	1.37	1.24 (0.23)	0.87	2
5.237	Glucose-6-phosphate*	1.38E-03	1.57	1.54 (0.33)	0.91	2
4.648	Glucose-6-phosphate*	1.89E-03	1.54	-1.49 (0.96)	0.92	2
4.658	Glucose-6-phosphate*	2.29E-03	1.51	-1.48 (0.98)	0.94	2
5.244	Glucose-6-phosphate*	2.47E-03	1.51	1.46 (0.46)	0.84	2
4.002	Glucose-6-phosphate*	3.54E-02	1.12	1.14 (0.42)	0.80	2
2.885	Asparagine	1.62E-03	1.55	1.25 (0.31)	0.94	1
4.002	Asparagine*	3.54E-02	1.12	1.14 (0.42)	0.80	1
2.177	Acetylcholine	2.43E-03	1.51	-1.32 (0.31)	0.89	1
2.545	Citric acid*	2.49E-03	1.51	-1.49 (0.45)	0.92	1S
2.535	Citric acid*	8.23E-03	1.36	-1.39 (0.21)	0.86	1S
2.553	Citric acid*	1.57E-02	1.26	-1.31 (0.31)	0.82	1S
7.096	Carnosine	2.52E-03	1.50	1.6 (0.75)	0.82	2
1.930	Arginine*	2.62E-03	1.50	1.24 (0.23)	0.84	2
1.690	Arginine*	3.04E-02	1.15	-1.17 (0.56)	0.81	2
1.697	Arginine*	3.49E-02	1.12	-1.17 (0.52)	0.77	2
3.829	Fructose*	2.93E-03	1.49	1.24 (0.29)	0.87	2

3.689	Fructose	2.63E-02	1.17	1.1 (0.2)	0.83	2
3.726	Fructose	2.70E-02	1.17	1.17 (0.33)	0.79	2
3.562	Fructose	3.53E-02	1.12	1.15 (0.35)	0.78	2
4.002	Fructose*	3.54E-02	1.12	1.14 (0.42)	0.80	2
3.714	Fructose	3.87E-02	1.10	1.15 (0.21)	0.81	2
3.943	Creatine	3.32E-03	1.47	-1.21 (0.65)	0.89	3S
2.504	Dimethylamine	3.35E-03	1.47	1.9 (0.59)	0.84	1S
0.881	Capric acid	8.83E-03	1.35	-1.27 (0.4)	0.79	1
1.276	Capric acid	4.57E-02	1.07	-1.17 (0.32)	0.76	1
6.903	Tyramine	1.17E-02	1.30	1.25 (0.42)	0.80	2
6.912	Tyramine	1.69E-02	1.25	1.26 (0.42)	0.82	2
7.195	Tyramine	2.96E-02	1.15	1.19 (0.47)	0.77	2
5.189	Mannose-6-phosphate	1.25E-02	1.29	0.25 (9.62)	0.81	2
5.902	Uridine	1.25E-02	1.29	3.63 (3.13)	0.78	2
5.911	Uridine	2.66E-02	1.17	2.02 (1.61)	0.78	2
5.922	Uridine	7.93E-02	0.95	1.7 (2.21)	0.67	2
5.812	Uracil	1.84E-02	1.23	1.8 (0.8)	0.80	1
5.803	Uracil	2.47E-02	1.18	1.75 (0.94)	0.79	1
1.254	3-Hydroxyisovaleric acid	1.92E-02	1.23	-1.68 (2.74)	0.97	1S
1.490	Alanine	2.64E-02	1.17	1.16 (0.16)	0.79	3
3.706	Tagatose	3.58E-02	1.12	-1.13 (0.28)	0.79	2
1.337	Lactic acid*	3.97E-02	1.10	1.13 (0.27)	-0.23	3
1.993	Acetamide	3.93E-02	1.10	1.13 (0.23)	0.81	1S
0.992	Valine	4.34E-02	1.08	1.16 (0.37)	0.77	1
1.000	Valine	4.50E-02	1.07	1.15 (0.39)	0.79	1
3.948	Galactaric acid	5.02E-02	1.05	-1.12 (0.52)	0.80	2

Table S6. Full list of unidentified buckets for AsPC-1 that were determined to be significant.

ppm	p-value	VIP	Fold Change (Error)	AUC
1.187	6.62E-08	2.10	-2.62 (0.67)	1.00
0.173	2.03E-06	1.99	-8.22 (3.46)	1.00
4.492	3.78E-06	1.97	-2.62 (1.52)	1.00
4.500	3.97E-06	1.96	-2.78 (1.51)	1.00
0.067	1.33E-04	1.77	-2.4 (0.81)	0.96
4.422	3.47E-04	1.69	-1.35 (0.29)	0.94
3.470	3.78E-04	1.69	1.36 (0.48)	0.93
0.167	4.35E-04	1.68	-1.57 (1)	0.94
2.983	7.34E-04	1.63	-1.32 (0.36)	0.92
1.033	8.13E-04	1.62	-1.83 (1.03)	0.88
4.445	9.76E-04	1.60	-1.45 (0.77)	0.93
1.640	1.79E-03	1.54	1.32 (0.17)	0.90
2.185	2.65E-03	1.50	-1.32 (0.29)	0.88
4.381	2.70E-03	1.50	-1.24 (0.47)	0.90
1.147	2.77E-03	1.49	-2.05 (0.63)	0.88
1.076	3.95E-03	1.45	-1.39 (0.47)	0.82
5.414	4.53E-03	1.43	2.03 (0.89)	0.86
1.068	5.19E-03	1.42	-1.29 (0.72)	0.87

4.331	5.40E-03	1.41	-1.21 (0.54)	0.86
2.578	5.56E-03	1.41	-1.4 (0.35)	0.87
3.992	6.92E-03	1.38	1.2 (0.49)	0.84
2.570	9.13E-03	1.34	-1.32 (0.43)	0.82
2.561	1.08E-02	1.32	-1.35 (0.32)	0.83
-0.072	1.73E-02	1.24	-2.03 (1.37)	0.81
2.397	1.96E-02	1.22	-1.26 (0.93)	0.74
2.772	2.40E-02	1.19	-1.17 (0.24)	0.77
3.225	2.72E-02	1.17	-1.16 (0.23)	0.79
1.844	3.12E-02	1.14	1.19 (0.31)	0.77
1.110	3.47E-02	1.12	-1.52 (2.63)	0.77
2.160	3.91E-02	1.10	-1.16 (0.24)	0.73
7.684	4.07E-02	1.09	180 (257)	0.81
4.118	4.19E-02	1.09	1.31 (0.49)	0.74
2.104	4.49E-02	1.07	-1.14 (0.64)	0.82
2.146	4.82E-02	1.06	1.13 (0.3)	0.78
1.479	5.91E-02	1.02	1.15 (0.19)	0.73
3.933	6.13E-02	1.01	-1.12 (0.5)	0.80
3.680	6.18E-02	1.01	1.1 (0.22)	0.80
2.164	6.30E-02	1.00	-1.19 (0.63)	0.70
3.190	6.46E-02	1.00	-1.21 (0.42)	0.73
3.579	6.62E-02	0.99	-1.13 (0.32)	0.72

2.416	6.90E-02	0.98	-1.16 (0.52)	0.70
1.258	7.27E-02	0.97	-1.15 (0.4)	0.73
7.204	7.30E-02	0.97	1.17 (0.61)	0.73
7.339	7.54E-02	0.96	1.17 (0.83)	0.71
3.955	7.56E-02	0.96	-1.1 (0.34)	0.74
3.822	8.13E-02	0.95	1.1 (0.33)	0.78
2.141	8.59E-02	0.93	1.11 (0.3)	0.74
1.329	8.77E-02	0.93	1.11 (0.3)	0.74
2.756	8.93E-02	0.92	-1.13 (0.3)	0.77
2.349	9.06E-02	0.92	1.1 (0.29)	0.73
2.357	9.23E-02	0.92	1.09 (0.28)	0.70
3.139	9.62E-02	0.91	-1.2 (0.46)	0.70
4.032	9.99E-02	0.90	-1.11 (0.61)	0.72
1.705	1.00E-01	0.90	-1.12 (0.59)	0.70
6.152	1.01E-01	0.89	1.61 (1.01)	0.72
3.197	1.04E-01	0.89	-1.22 (0.53)	0.70
7.432	1.08E-01	0.88	1.15 (0.42)	0.74
3.719	1.11E-01	0.87	1.12 (0.37)	0.76
2.153	1.13E-01	0.87	1.11 (0.32)	0.73
3.755	1.14E-01	0.87	1.07 (0.2)	0.78
3.339	1.16E-01	0.86	-1.12 (0.65)	0.70
2.377	1.22E-01	0.85	-1.12 (0.31)	0.72

3.841	1.46E-01	0.80	1.1 (0.43)	0.72
3.072	1.57E-01	0.78	-1.09 (0.44)	0.73
3.762	1.78E-01	0.75	1.06 (0.24)	0.76
7.441	1.78E-01	0.75	1.19 (0.53)	0.71
1.051	1.83E-01	0.74	1.09 (0.33)	0.72
3.588	1.85E-01	0.73	1.09 (0.27)	0.70
2.856	2.20E-01	0.68	-1.12 (0.41)	0.71
3.527	2.59E-01	0.63	1.19 (0.72)	0.76
4.101	3.55E-01	0.52	1.57 (1.07)	0.72
1.312	5.20E-01	0.36	-1.08 (0.45)	0.58
